# The claudin family characteristics in pan-cancer and the role of claudin12 in the malignant progression of lung adenocarcinoma

**DOI:** 10.1038/s41598-025-28317-7

**Published:** 2025-12-29

**Authors:** Pei Gao, Yan Wu, Yuxin Bian, Yixuan Liu, Weixin Jing

**Affiliations:** 1 Department of Biology, School of Basic Medical Sciences, Xinjiang Medical University, Urumqi, 830017 China; 2 Clinical Laboratory Center, the Fourth Affiliated Hospital of Xinjiang Medical University, Urumqi, 830001 China; 3 Xinjiang Key Laboratory of Molecular Biology for Endemic Diseases, Xinjiang Medical University, Urumqi, 830017 China

**Keywords:** Pan-cancer, Claudin 12, Lung adenocarcinoma, Migration, Invasion, Cancer, Biomarkers

## Abstract

Claudins (CLDNs), pivotal components of tight junctions (TJs), are integral to epithelial barrier function, cell polarity, and paracellular permeability as well as in tumor initiation and development. Among the histological subtypes of lung cancer, lung adenocarcinoma (LUAD) predominates and is frequently diagnosed at an advanced stage, contributing to its dismal 5-year survival rate and posing a significant threat to patient survival. In this work, the expression, prognosis and diagnostic of CLDNs in pan-cancer were analyzed using bioinformatics. In particular, the relationship between claudin12 (CLDN12) and the expression, prognosis and clinical stage in LUAD was paid attention to, and the diagnostic efficiency of CLDN12 was evaluated by receiver operator characteristic curve. The PPI network was constructed using STRING database, followed by Cytoscape-assisted hub gene identification. Functional enrichment analyses (GO and KEGG) were conducted to elucidate potential signaling pathways linked to CLDN12. These results showed that CLDNs were differentially expressed in pan-cancer, and were valuable for the prognosis and diagnosis of tumors. Specifically, CLDN12 was significantly upregulated in LUAD, correlating with advanced tumor stage, lymph nodes metastasis, and poor survival outcomes. In vitro functional assays revealed that CLDN12 knockdown suppressed LUAD cell proliferation, migration, and invasion while promoting cell apoptosis. In summary, CLDN12 may serve as a promising biomarker for LUAD detection and a potential therapeutic target.

**Supplementary Information:**

The online version contains supplementary material available at 10.1038/s41598-025-28317-7.

## Introduction

Malignant neoplasms constitute a formidable challenge to global public health systems and socioeconomic stability. Among these, pulmonary malignancies represent the predominant oncologic pathology worldwide, responsible for approximately 18.7% of total cancer mortality^1^. The epidemiological burden is particularly pronounced in China, where lung cancer contributes to nearly 30% of cancer-associated fatalities^2^. Projection analyses indicate that lung cancer will remain one of the five most prevalent oncologic entities in China during the 2028–2032 surveillance period^3^. Lung adenocarcinoma (LUAD) accounts for 40 to 45% of lung cancer cases^4^. Due to the lack of early symptoms of LUAD, patients often have no obvious respiratory symptoms resulting in late-stage diagnosis in approximately two-thirds of cases (stage III/IV)^5^. Notably, LUAD is characterized by easy invasion and metastasis, the 5-year survival rate for patients is low, posing significant threats to global health security^6^. More than 90% of human cancer deaths are caused by tumor cell metastasis^7^. Given these clinical realities, elucidating the molecular underpinnings of LUAD progression and metastatic spread represents a critical research priority with substantial translational implications.

As specialized intercellular connections, tight junctions (TJs) constitute the apical-most component of junctional complexes in epithelial and endothelial cellular layers. These macromolecular structures serve multiple critical functions, such as maintaining cell barriers, regulating material transport, preserving cell polarity, and participating in intracellular signal transduction^8^. In epithelial cells, the primary functions of TJs are to regulate the adhesion between cells and extracellular matrix^9^. Alterations in TJs expression can compromise intercellular adhesive integrity, representing a critical pathophysiological mechanism in malignant transformation and carcinogenesis^10^. The malignant progression of neoplasms is fundamentally mediated through three critical biological processes: cellular migration, tissue invasion, and distant metastasis. Successful metastatic dissemination requires tumor cells to undergo full epithelial mesenchymal transition (EMT), that is, dissolution of apical-basal polarity, disintegration of intercellular junctions (particularly TJs), and to activate the ability to invade and enter the vasculature^11^. TJs serve as a pivotal role in activating cancer invasion and metastasis, as loss of intercellular junctions is a prerequisite^12^. Claudins (CLDNs) are the structural basis of TJs^7^, consisting of cytoplasmic N-and C-terminus, four transmembrane domains, two extracellular loops and one intracellular loop domain^13^. CLDNs exhibit distinct tissue- and development-dependent distribution profiles, and are detectable in both epithelial and endothelial cell types. Abnormal expression of CLDNs can change the normal structure of TJs, and cause the inhibition of intercellular contact and forfeiture of cellular polarity, and enhance the ability of cell invasion and metastasis^14^. Simultaneously, CLDNs possess tissue-specific and tumor-specific regulatory mechanisms. They engage in modulating diverse signaling cascades across various neoplastic and pathological conditions. CLDNs are intricately associated with the invasive and metastatic processes of multiple malignant tumors, and have thus been exploited as potential targets for tumor diagnosis or employed as prognostic indicators^15^. For example, CLDN10 exhibits elevated expression in high-grade serous carcinoma, and thus may function as a novel prognostic biomarker for this malignancy^16^. CLDN1 demonstrates overexpression in esophageal squamous cell carcinoma. It initiates autophagy via the AMPK/STAT1/ULK1 signaling axis, thereby facilitating the proliferation and metastasis of esophageal squamous cell carcinoma^17^.

Claudin12 (CLDN12) is located at 7q21 and has two introns encoding 244 amino acids with a molecular weight of 27 kDa^18^. Analogous to other CLDNs, CLDN12 exhibits significant biological functions related to the permeability of epithelial and epidermal tissues, barrier safeguarding, and cellular junctions. Unlike other CLDNs, CLDN12 does not have a PDZ domain that interacts with the cytoskeleton^19^. CLDN12 is the most widely expressed gene in the CLDN family and is constitutively expressed in most tissues^20^, while showing the most prominent expression in renal tissues. Currently, the comprehension of CLDN12’s biological functions predominantly centers on its involvement in epithelial and epidermal permeability and cell junction regulation. For instance, CLDN12 acts as a barrier between cells in tight junction^21^. It is capable of forming a calcium-selective channel in intestinal epithelial cells, playing a crucial role in the process of calcium absorption^22^. In addition, studies on CLDN12 and tumors are also increasing. In the study of mouse skin tumors, the amount of CLDN12 in the suprabasal cortex decreases with tumorigenesis and tumor progression^23^. CLDN12 is expressed in human colon adenocarcinoma (COAD) LS180 cells and exerts a pivotal influence on the dissemination of cancer cells^24^. However, studies on CLDN12 and LUAD are very limited. As the predominant histological subtype of lung cancer, LUAD is still a major global medical challenge, primarily attributed to its elevated incidence and mortality rates^25^. Consequently, there exists an imperative requirement to identify safer and more reliable biomarkers for the early detection and targeted treatment of LUAD, thereby alleviating the suffering and burden endured by patients.

In the present investigation, bioinformatics approaches were employed to assess the expression profiles and prognostic associations of CLDNs across pan-cancer datasets. Particular emphasis was placed on elucidating the expression patterns, prognostic significance, and clinical correlations of CLDN12 in LUAD. The diagnostic efficiency of CLDN12 was evaluated by receiver operating characteristic (ROC) curve. The STRING database was used to construct the protein-protein interaction (PPI) network, and Cytoscape and different plugins were used to calculate and screen out the hub genes. Gene Ontology (GO) and Kyoto Encyclopedia of Genes and Genomes (KEGG) analyses were conducted to investigate the potential associated pathways of CLDN12. Then, the role of CLDN12 in the proliferation, migration, invasion and apoptosis of LUAD was verified by in vitro experiments, which provided new ideas for the early diagnosis and clinical treatment of LUAD.

We have found that CLDNs exhibit differential expression in various normal tissues and different types of cancers, and they are of great significance in the diagnosis and prognosis of cancer. Interestingly, CLDN12 is highly expressed in most types of cancer. We are particularly interested in its relationship with LUAD. CLDN12 is upregulated in LUAD and is associated with the tumor stage and lymph nodes metastasis of LUAD patients. Knockdown of CLDN12 inhibits the proliferation, migration and invasion of LUAD cells, while promoting their apoptosis.

## Results

### CLDNs in pan-cancer

**Fig. 1:**
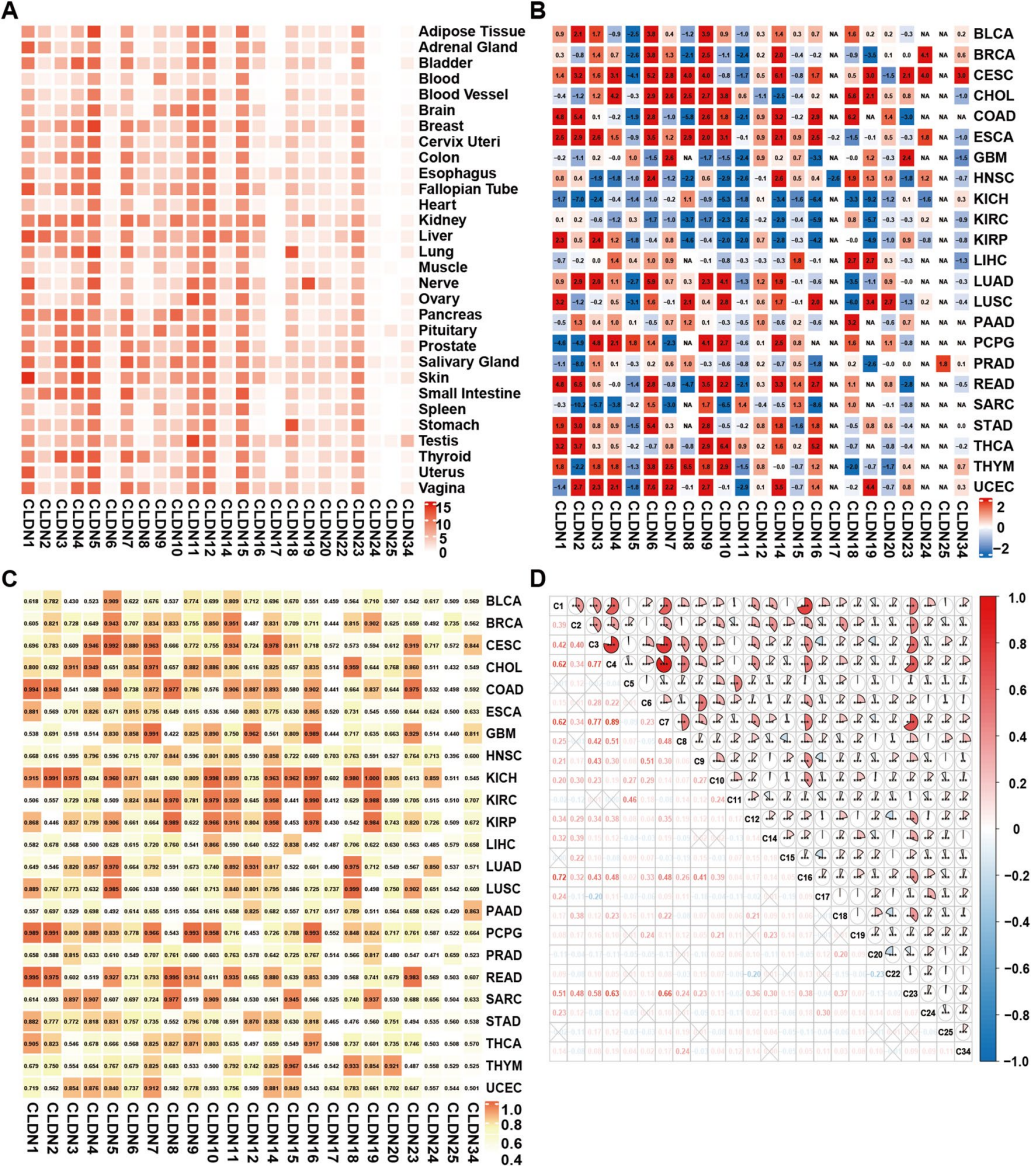
Expression of CLDNs in pan-cancer. (**A**) CLDNs expression in GTEx normal tissues; The color scale represents log_2_(expected count + 1). (**B**) Differential expression of CLDNs in TCGA cancer tissues; The color scale represents log_2_(FC). (**C**) Heatmap of AUC value of CLDNs in pan-cancer of TCGA database. (**D**) Correlation heatmap of CLDNs expression in pan-cancer in TCGA database. In the figure, C represents CLDNs, and the numbers represent the correlation coefficients. This figure was generated using the ComplexHeatmap package (version 2.22.0), pROC package (version 1.18.5), and corrplot package (version 0.95) in R (version 4.3.0). Graphic stitching was performed using Adobe Illustrator (version 24.3.0).

As shown in Fig. 1A, data from the Genotype-Tissue Expression (GTEx) database demonstrate that the expression of CLDNs in normal tissues exhibits tissue specificity, with varying expression levels among different CLDNs. Specifically, CLDN1, 5, 11, 12, 15, and 23 are predominantly highly expressed across most normal tissues, whereas CLDN6, 17, 24, 25, and 34 display low or undetectable expression in the majority of tissues. As shown in Fig. 1B, analysis of The Cancer Genome Atlas (TCGA) database reveals that nearly all CLDNs display distinct expression profiles across diverse tumor types. For instance, CLDN1 and CLDN2 are significantly upregulated in COAD, with log_2_FC of 4.8 and 5.4, respectively. However, CLDN1 shows marked downregulation in pheochromocytoma and paraganglioma (PCPG) (log_2_FC = -4.7), and CLDN2 exhibits even more pronounced downregulation in sarcoma (SARC) (log_2_FC = -10.2). The expression levels of CLDN5, 11, and 23 did not exhibit significant variations across the majority of tumor types. In kidney chromophobe (KICH), kidney renal clear cell carcinoma (KIRC), and kidney renal papillary cell carcinoma (KIRP), the expression of CLDNs was predominantly downregulated. For CLDN1 and CLDN15, significant differential expression was observed in certain tumors, whereas no such differences were noted in others. Notably, CLDN12 displayed differential expression patterns across most tumor entities, with particularly pronounced upregulation in LUAD (log_2_FC = 1.2). Many similar results suggest that different CLDNs play distinct roles in different tumors^26–29^. Moreover, significant heterogeneity in CLDNs expression was evident when comparing different histological subtypes of tumor tissues. The results of GTEx integration of TCGA database showed that most of the CLDNs were differentially expressed in 33 types of cancer (Figure S1).

The ROC for CLDNs were generated across 33 types of cancer in the TCGA database, and the corresponding areas under the curve (AUC) were computed. The analysis revealed that the AUC for CLDNs exceeded 0.7 in the majority of tumors, with certain values reaching above 0.9 (Fig. 1C). A correlation heatmap depicting CLDNs expression patterns across pan-cancer datasets demonstrated significant positive correlations (*P* < 0.05) among most CLDN family members. Notably, CLDN4 and CLDN7 exhibited the highest correlation coefficient, measuring 0.89 (Fig. 1D). As shown in Figure S2, univariate COX proportional hazards regression analyses of CLDNs were conducted across 33 types of cancer within the TCGA database. The results indicated that CLDNs expression levels served as independent prognostic factors for patient survival in diverse cancer contexts (*P* < 0.05).

The mutation landscape of LUAD retrieved from the cBioPortal online database showed that, among LUAD cases, the proportion of “mRNA high” is the highest among all CLDN family gene alteration types, accounting for nearly 50%. In contrast, other modification types, including gene mutations, amplifications, deep deletions, and multiple compound alterations, occur at lower frequencies (Figure S3A). Specifically, the alteration frequency of CLDN4 in LUAD patients is 12%, while that of CLDN12 is 9% (Figure S3B).

### CLDN12 in pan-cancer

**Fig. 2:**
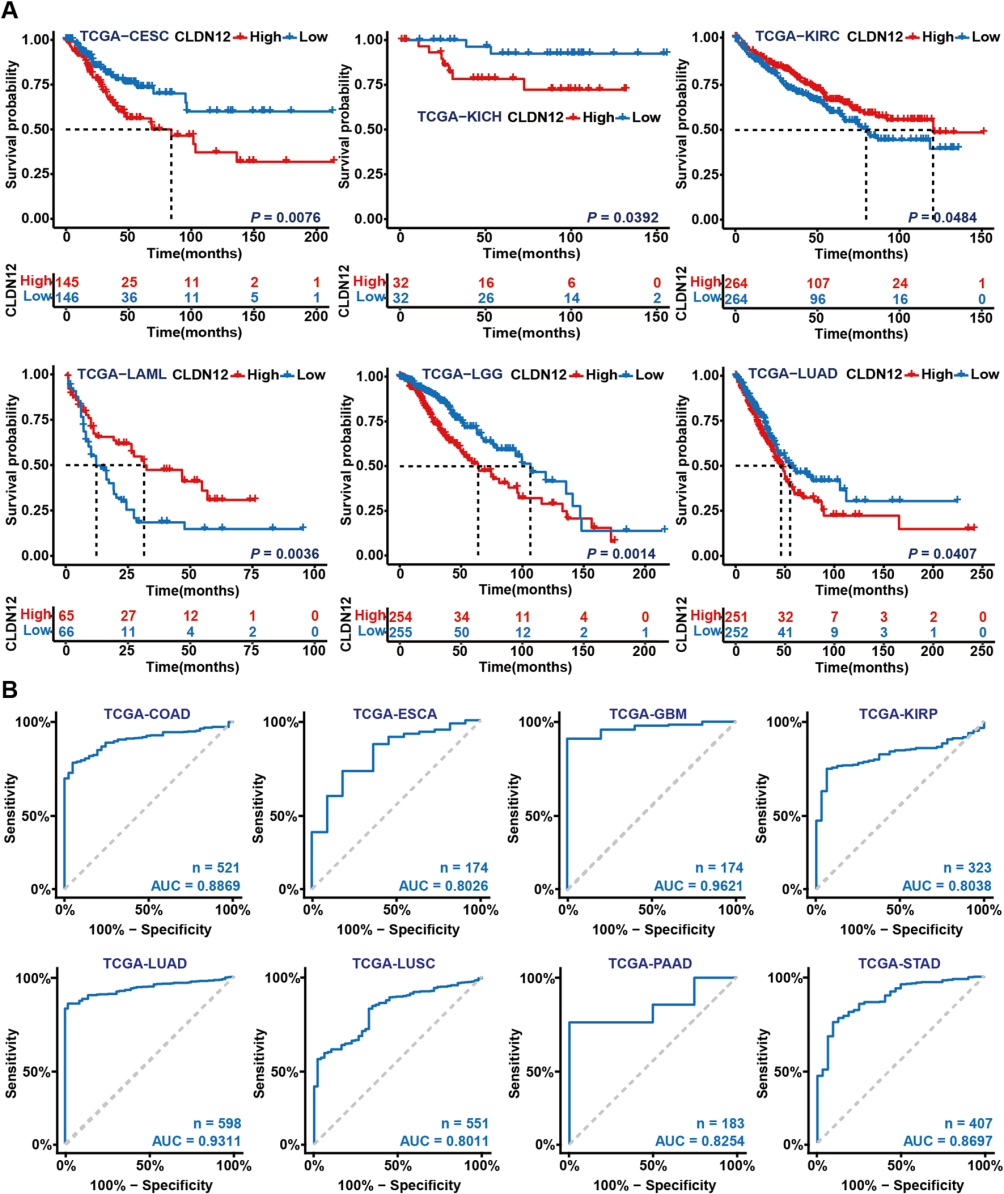
Association analysis of CLDN12 in pan-cancer. (**A**) KM survival curve of CLDN12 in TCGA database. (**B**) ROC curve of CLDN12 in TCGA database. This figure was generated using the survival package (version 3.5-7), survminer package (version 0.4.9), pROC package (version 1.18.5) in R (version 4.3.0). Graphic stitching was performed using Adobe Illustrator (version 24.3.0).

The integration of 33 types of cancer in GTEx and TCGA databases showed that CLDN12 expression was significantly different in most cancers compared with normal tissues (*P* < 0.05) (Figure S1). In TCGA database tumor paired samples, compared with normal tissues, the expression of CLDN12 in bladder urothelial carcinoma (BLCA), COAD, esophageal carcinoma (ESCA), KICH, hepatocellular carcinoma (LIHC), lung squamous cell carcinoma (LUSC), KIRC, prostate adenocarcinoma (PRAD), rectal adenocarcinoma (READ), stomach adenocarcinoma (STAD) and KIRP tumor tissues was significantly different (*P* < 0.05) (Figure S4). Immunohistochemistry (IHC) of tumor samples and normal tissues from the Human Protein Atlas (HPA) database revealed that CLDN12 protein was highly expressed in LUSC, PRAD, COAD, READ, LIHC, cholangiocarcinoma (CHOL), breast invasive carcinoma (BRCA), and BLCA tumor tissues (Figure S5). The Kaplan-Meier (KM) curve depicted in Fig. 2A shows the correlation between the expression level of CLDN12 and the survival rate of cancer patients. A high expression of CLDN12 was found to be associated with a poor prognosis in patients with LUAD, cervical squamous cell carcinoma and endocervical adenocarcinoma (CESC), KICH and lower-grade glioma (LGG). Conversely, in patients with KIRC and acute myeloid leukemia (LAML), a high CLDN12 expression was linked to a better prognosis (*P* < 0.05). Next, we plotted the ROC with AUC greater than 0.8 for CLDN12 in pan-cancer (Fig. 2B) and found that it was largest in glioblastoma multiforme (GBM) at 0.9621, followed by LUAD at 0.9311.

### CLDN12 in LUAD

**Fig. 3:**
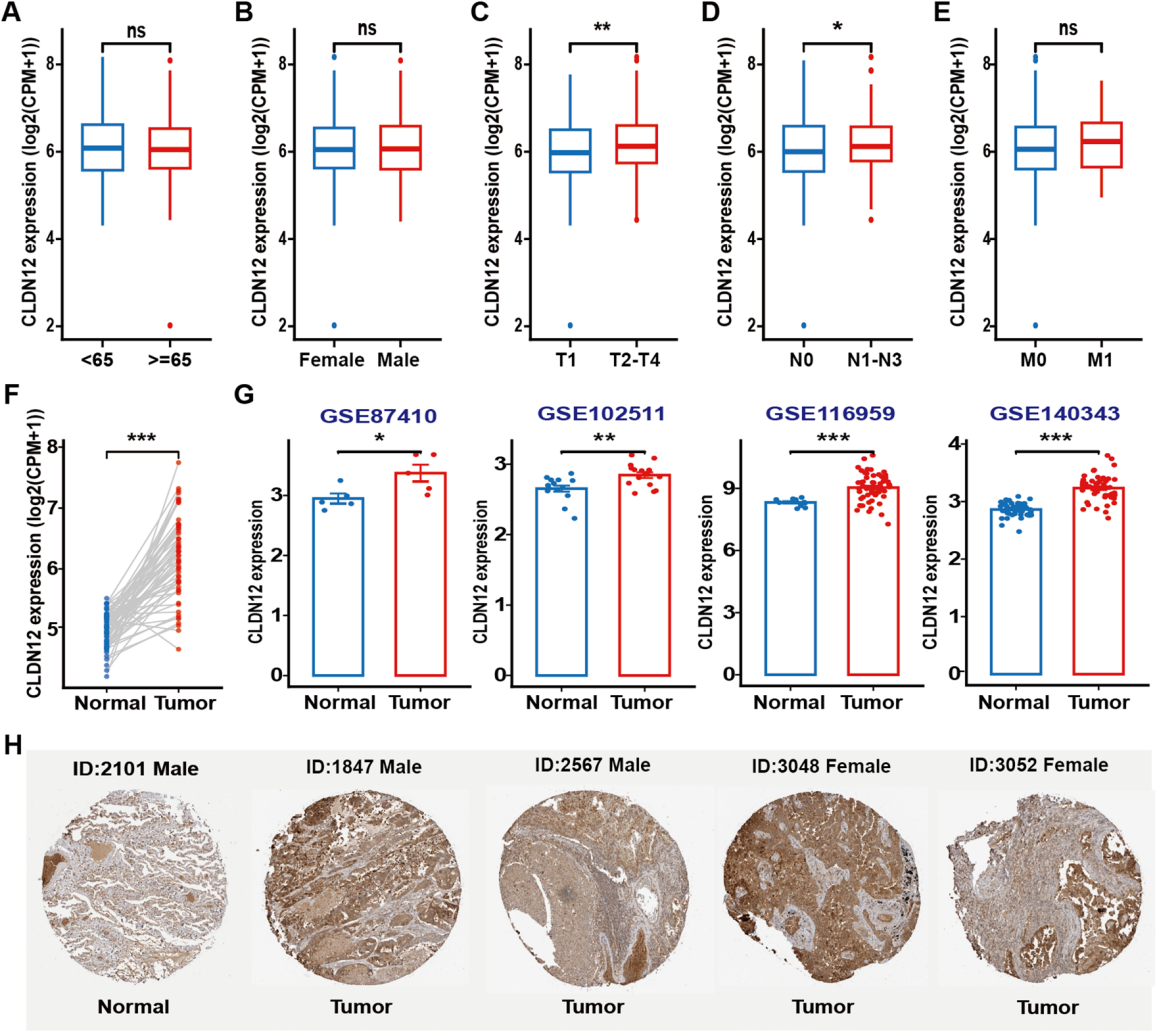
Correlation between CLDN12 and clinical features of LUAD and expression of CLDN12 in LUAD. Correlation between CLDN12 and LUAD clinical features in TCGA database: (**A**) age, (**B**) gender, (**C**) TNM stage, (**D**) lymph nodes metastasis, (**E**) distant metastasis. (**F**) CLDN12 expression in LUAD paired samples from TCGA database. (**G**) Expression analysis of CLDN12 in four LUAD datasets in GEO database. (**H**) Protein expression of CLDN12 in HPA database in normal lung tissue and LUAD samples. (*P < 0.05, **P < 0.01, ***P < 0.001). This figure was generated using the ggplot2 package (version 3.5.1) in R (version 4.3.0). Graphic stitching was performed using Adobe Illustrator (version 24.3.0).

As shown in Fig. 3C and D, the expression levels of CLDN12 in T2-T4 and N1-N3 stages were significantly higher than those in the control group (*P* < 0.05). In contrast, no significant differences in CLDN12 expression were observed among various clinical characteristics of LUAD, including age, gender, and the presence of distant metastasis, as shown in Fig. 3A and B, and 3E. In TCGA database paired samples, CLDN12 expression was significantly upregulated in LUAD compared with adjacent normal tissues (*P* < 0.05) (Fig. 3F). As in the previous results, CLDN12 levels in the GTEx and TCGA databases were significantly higher in LUAD (*P* < 0.05) (Figure S1). We validated four LUAD datasets in the Gene Expression Omnibus (GEO) database, which also showed that CLDN12 was highly expressed in LUAD (*P* < 0.05) (Fig. 3G). IHC data from the HPA database showed that CLDN12 protein levels were upregulated in LUAD compared with normal lung tissue (Fig. 3H).

### Functional enrichment analysis of genes related to CLDN12 expression

**Fig. 4:**
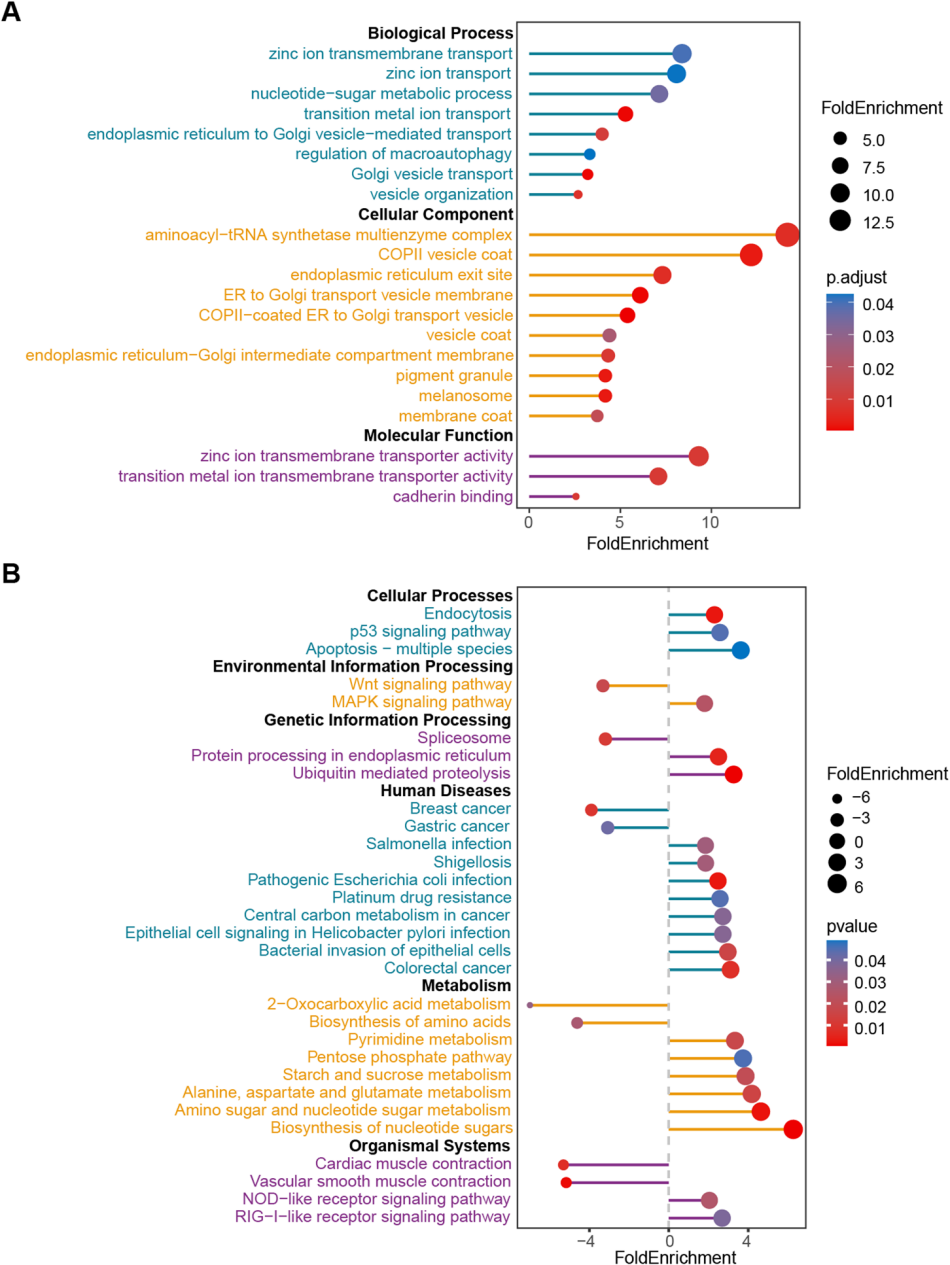
Analysis of genes associated with CLDN12 expression. (**A**) GO functional enrichment analysis of related genes. (**B**) KEGG signaling pathway enrichment. This figure was generated using the clusterProfiler (version 4.14.4), enrichplot package (version 1.26.2) in R (version 4.3.0). Graphic stitching was performed using Adobe Illustrator (version 24.3.0).

GO functional enrichment analysis demonstrated that genes associated with CLDN12 expression were implicated in biological processes including transition metal ion transport, vesicle organization, and endoplasmic reticulum to Golgi vesicle-mediated transport. It is involved in the composition of cellular components such as aminoacyl-tRNA synthetase multienzyme complexes, endoplasmic reticulum to Golgi transport vesicles, and Coat Protein Complex II (COP II) vesicle capsids and plays a role in molecular functions such as zinc ion transmembrane transporter activity and cadherin (Fig. 4A). The KEGG pathway analysis revealed that genes related to CLDN12 expression were chiefly associated with endocytosis, apoptosis, p53 signaling pathway and other cellular processes. It is involved in the processing of genetic information such as spliceosome, protein processing in the endoplasmic reticulum, and ubiquitin-mediated proteolysis. It is involved in the biosynthesis of nucleotide sugars, closely related to the metabolism of starch, sucrose, and pyrimidines, and is associated with colorectal cancer, breast cancer, and gastric cancer. It was also enriched for myocardial contraction, vascular smooth muscle contraction, Wnt, MAPK, NOD-like receptor signaling pathway, and RIG-I-like receptor signaling pathway (Fig. 4B).

### CLDN12 PPI network analysis and hub gene screening

**Fig. 5:**
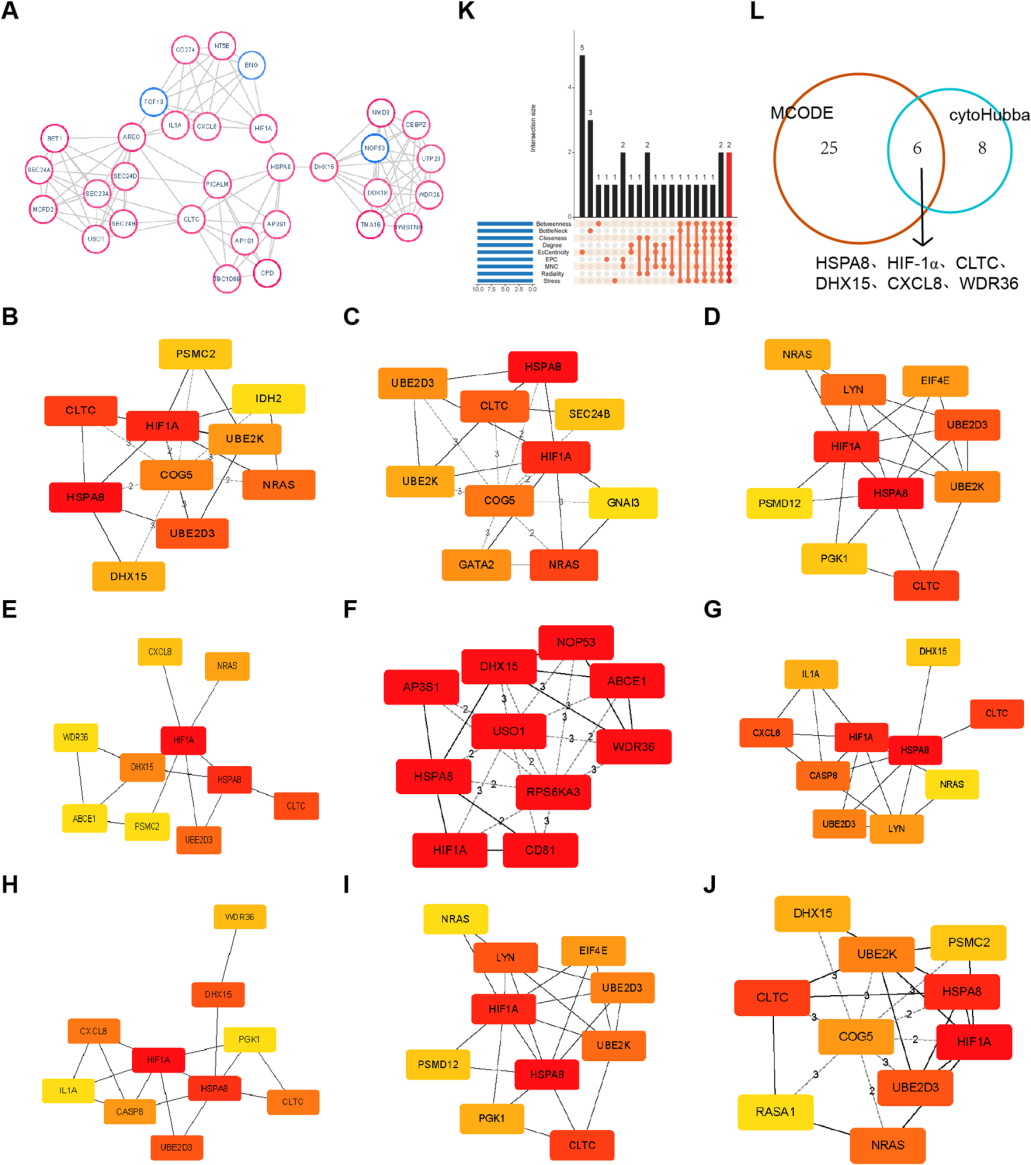
CLDN12 PPI network analysis. (**A**) The largest subnetwork screened by MCODE. (**B**-**J**) CytoHubba multi-algorithm core genes. (**K**-**L**) Core gene intersection. The PPI network was established and visualized using the STRING database (https://cn.string-db.org). Hub genes were analyzed and visualized using the CytoHubba plugin (version 0.1) and the MCODE plugin (version 1.6.1) in Cytoscape (version 3.7.0). Graphic stitching was performed using Adobe Illustrator (version 24.3.0).

The protein interaction network associated with CLDN12 was established by utilizing the STRING online database. After CLDN12 was eliminated, the PPI network diagram targeting CLDN12 was obtained (Figure S6). The network graph encompasses 635 nodes and 181 edges, and the genes shown in the graph are those with a connectivity degree greater than 15. The network map was imported into Cytoscape and hub genes were screened using the Mcode plugin, resulting in the subnetwork with the highest score (Fig. 5A), consisting of 31 genes. Nine topology algorithms integrated within the CytoHubba plugin were utilized to generate a core gene set. Each algorithm contributed its top 10 genes to this set (Fig. 5B-J). An analysis of the intersections among these 9 distinct algorithms from the CytoHubba plugin revealed that 14 genes were identified as core genes by three or more algorithms (Fig. 5K). Following this, an intersection operation was carried out between this set of core genes and the maximum subnetwork that had been previously screened using the Mcode plugin. As a consequence of this process, six hub genes were ultimately determined, namely heat shock protein family A (Hsp70) member 8 (HSPA8), hypoxia-inducible factor 1α (HIF-1α), clathrin heavy chain (CLTC), DEAH-box helicase 15 (DHX15), C-X-C motif chemokine ligand 8 (CXCL8), and WD repeat domain 36 (WDR36) (Fig. 5L).

### Expression of CLDN12 mRNA in LUAD cells and CLDN12 promoted the proliferation of LUAD cells

**Fig. 6:**
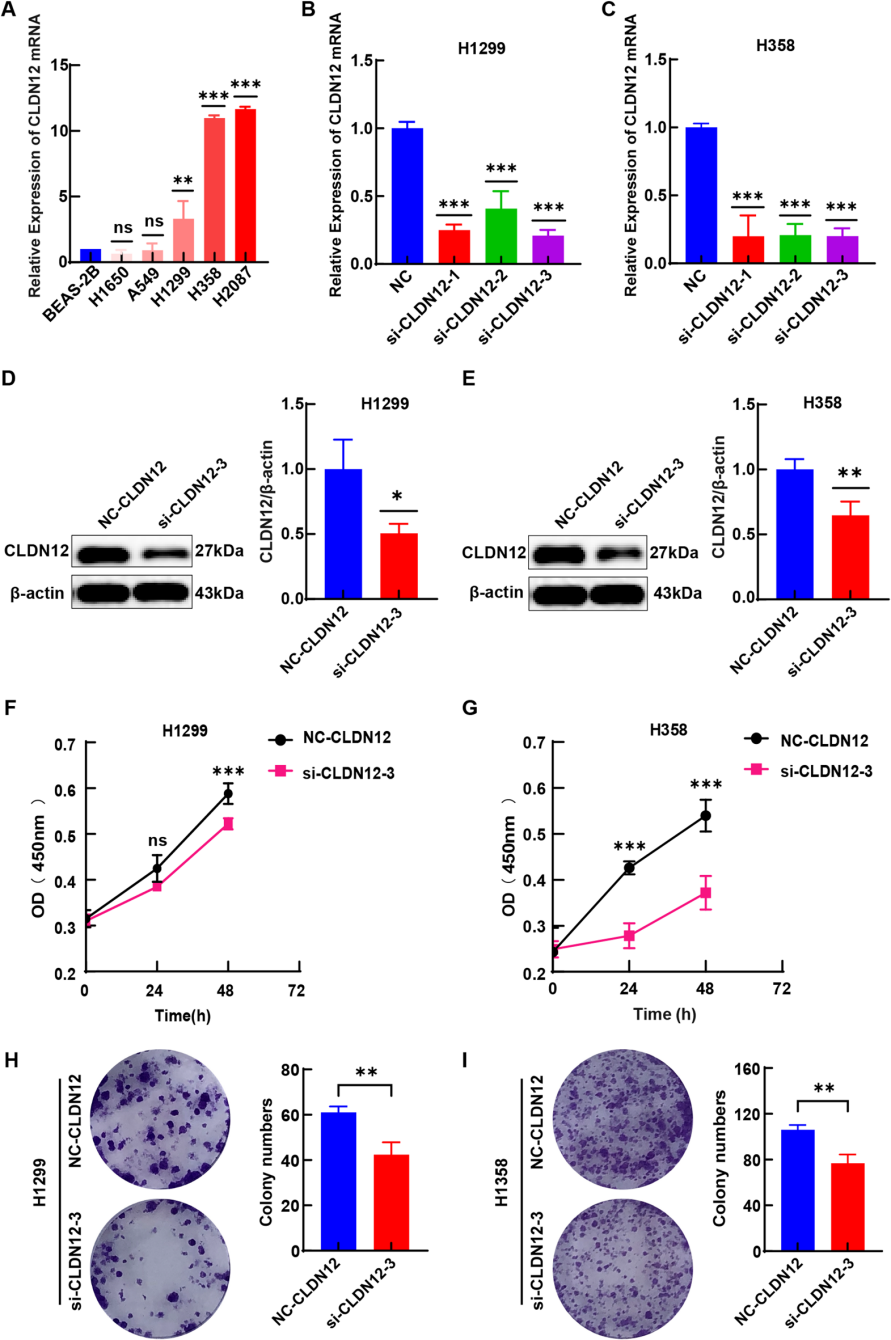
(**A**) CLDN12 mRNA expression in LUAD cells. (**B**,** C**) Downregulation of CLDN12 mRNA expression was confirmed by RT-qPCR. (**D**,** E**) Downregulation of CLDN12 protein expression was confirmed by WB (original images in Figure S7). (**F**-**I**) CLDN12 knockdown inhibited the proliferation of H1299 and H358 cells. (*P < 0.05, **P < 0.01, ***P < 0.001) ($\:\stackrel{-}{x}\pm\:s$, *n* = 3). This figure was generated using the GraphPad Prism (version 8.0). Graphic stitching was performed using Adobe Illustrator (version 24.3.0).

As shown in Fig. 6A, when contrasted with human normal bronchial epithelium cells BEAS-2B, the mRNA levels of CLDN12 were notably upregulated in H1299, H358, and H2087 cells (*P* < 0.05). However, no significant disparities in CLDN12 mRNA levels were observed between BEAS-2B cells and A549 as well as H1650 cells. Subsequently, we employed three si-RNA vectors for the specific knockdown of CLDN12. As validated by reverse transcription quantitative polymerase chain reaction (RT-qPCR), the expression of CLDN12 mRNA were significantly diminished in both H1299 and H358 cells (*P* < 0.05) (Fig. 6B and C). Among these vectors, si-CLDN12-3 demonstrated the most pronounced knockdown efficacy. Consequently, si-CLDN12-3 was ultimately chosen as the exclusive vector for the subsequent transient transfection aimed at silencing CLDN12. Subsequently, Western blotting (WB) results confirmed that the expression of CLDN12 protein was downregulated in H1299 and H358 cells (*P* < 0.05) (Fig. 6D and E). The cell counting kit-8 (CCK-8) assay results indicated that upon knockdown of CLDN12, the proliferation of H1299 and H358 cells was significantly suppressed after 48 h (*P* < 0.05) (Fig. 6F and G). Colony formation assay further corroborated that the knockdown of CLDN12 led to a notable inhibition of the colony-forming capacity of H1299 and H358 cells (*P* < 0.05) (Fig. 6H and I).

**Fig. 7:**
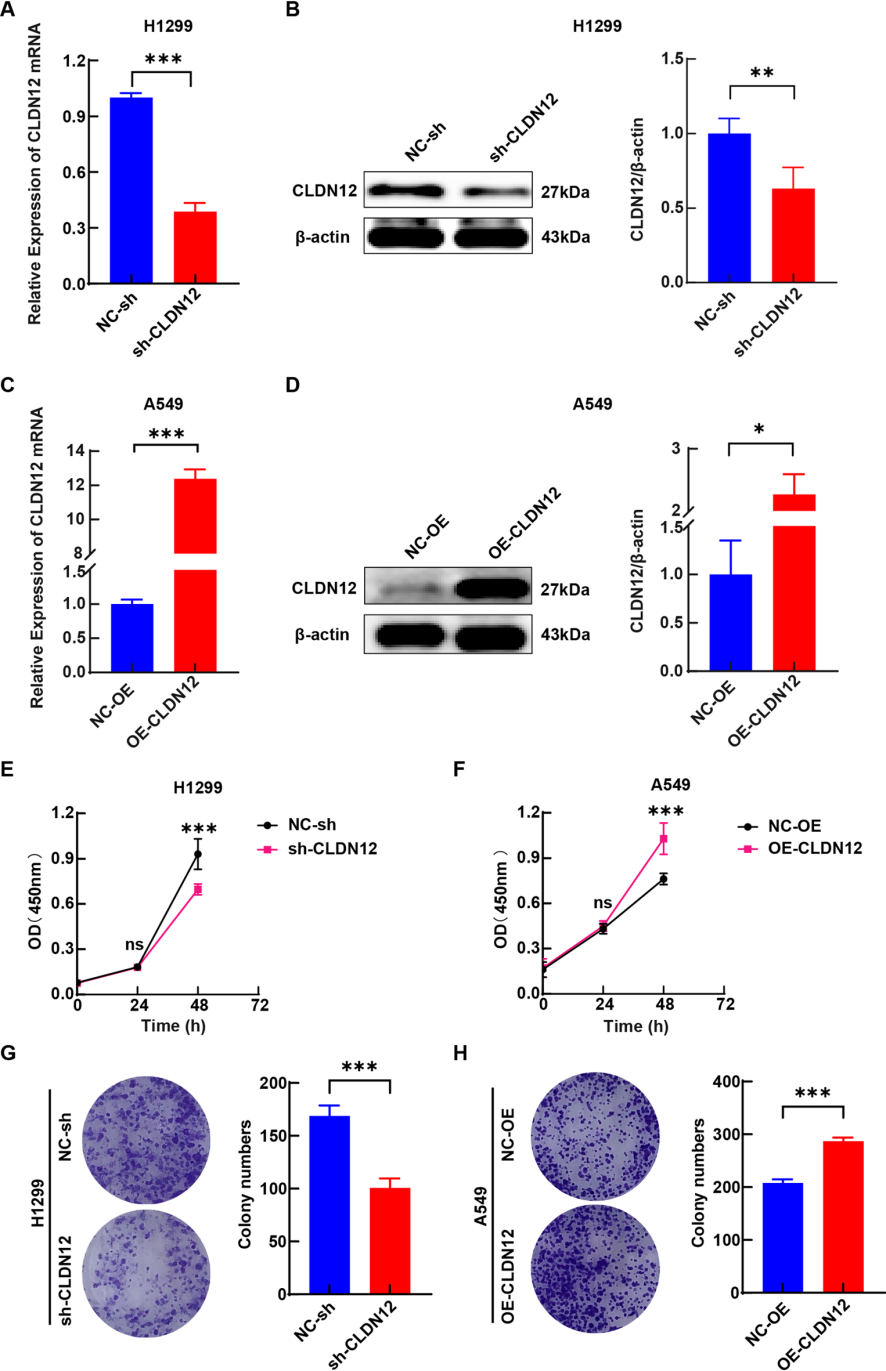
(**A**,** B**) Reduced expression of CLDN12 at the mRNA and protein levels was confirmed by RT-qPCR and WB (original images in Figure S8). (**C**,** D**) Increased expression of CLDN12 at the mRNA and protein levels was confirmed by RT-qPCR and WB (original images in Figure S9). (**E**,** G**) CLDN12 underexpression inhibited the proliferation of H1299 cells. (**F**,** H**) CLDN12 overexpression promoted the proliferation of A549 cells. (*P < 0.05, **P < 0.01, ***P < 0.001) ($\:\stackrel{-}{x}\pm\:s$, *n* = 3) (Note: Figure E: The 24-hour time point error bar is shorter than the data symbol, precluding visualization.). This figure was generated using the GraphPad Prism (version 8.0). Graphic stitching was performed using Adobe Illustrator (version 24.3.0).

Then, we constructed stable cell lines with CLDN12 underexpression and CLDN12 overexpression, respectively. As shown in Fig. 7A and B, in the H1299 cell line, the reduced expression of CLDN12 at the mRNA and protein levels was verified by RT-qPCR and WB, respectively (*P* < 0.05). As shown in Fig. 7C and D, in the A549 cell line, the increased expression of CLDN12 at the mRNA and protein levels was verified by RT-qPCR and WB, respectively (*P* < 0.05). Results from the CCK-8 assay showed that CLDN12 underexpression significantly suppressed the proliferation of H1299 cells at 48 h (*P* < 0.05), whereas CLDN12 overexpression significantly increased the proliferation of A549 cells at the same time point (*P* < 0.05) (Fig. 7E and F). Additionally, colony formation assay results demonstrated that CLDN12 underexpression significantly decreased the proliferative capacity of H1299 cells (*P* < 0.05), while CLDN12 overexpression significantly increased that of A549 cells (*P* < 0.05) (Fig. 7G and H).

### Knockdown of CLDN12 inhibits the migration and invasion of LUAD cells and promotes apoptosis

**Fig. 8:**
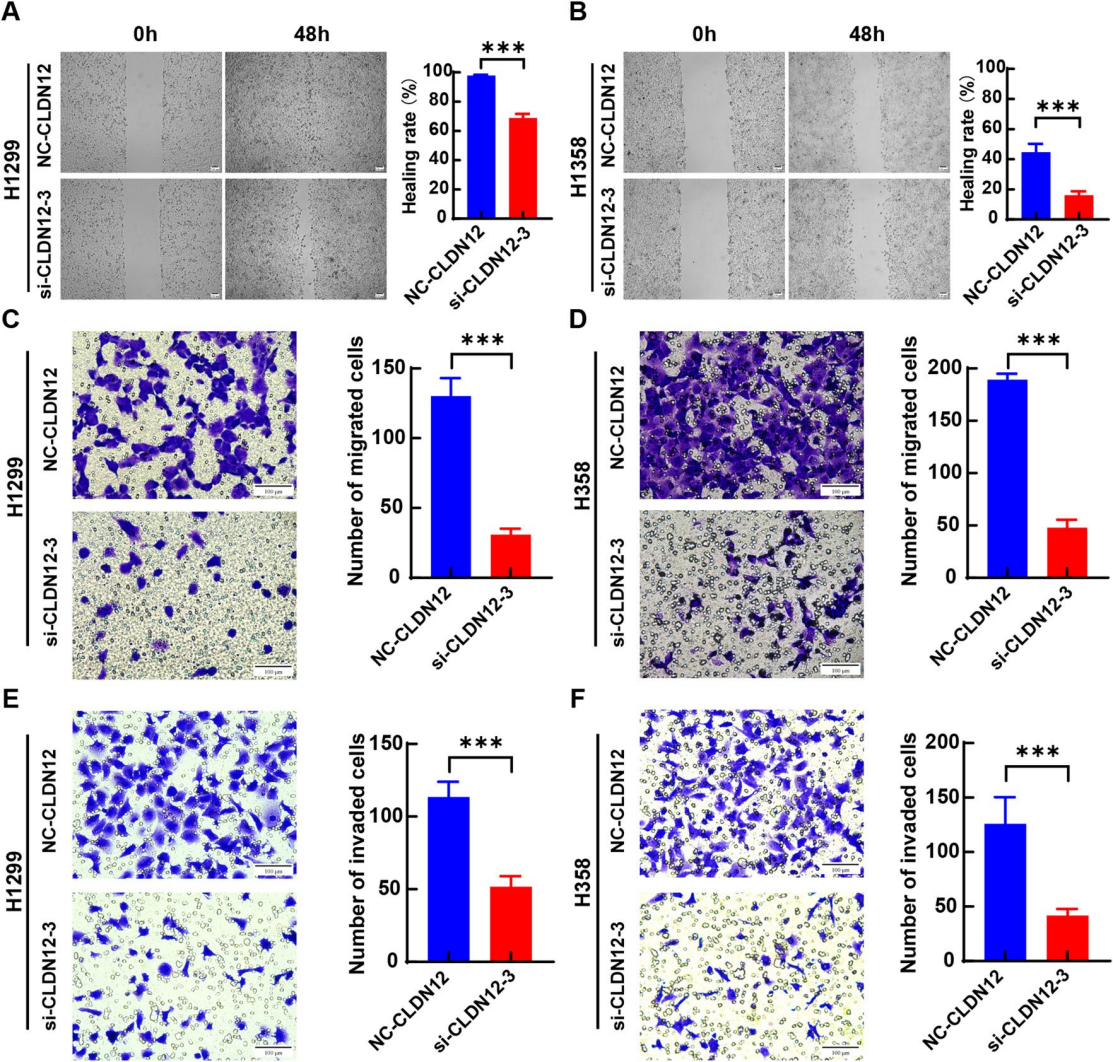
CLDN12 knockdown inhibited the migration and invasion of H1299 and H358 cells. (**A**,** B**) CLDN12 knockdown inhibited the wound healing ability of H1299 and H358 cells. (**C**,** D**) CLDN12 knockdown inhibited the migration of H1299 and H358 cells. (**E**,** F**) CLDN12 knockdown inhibited the invasion of H1299 and H358 cells. (***P < 0.001) ($\:\stackrel{-}{x}\pm\:s$, *n* = 3). This figure was generated using the GraphPad Prism (version 8.0). Graphic stitching was performed using Adobe Illustrator (version 24.3.0).

**Fig. 9:**
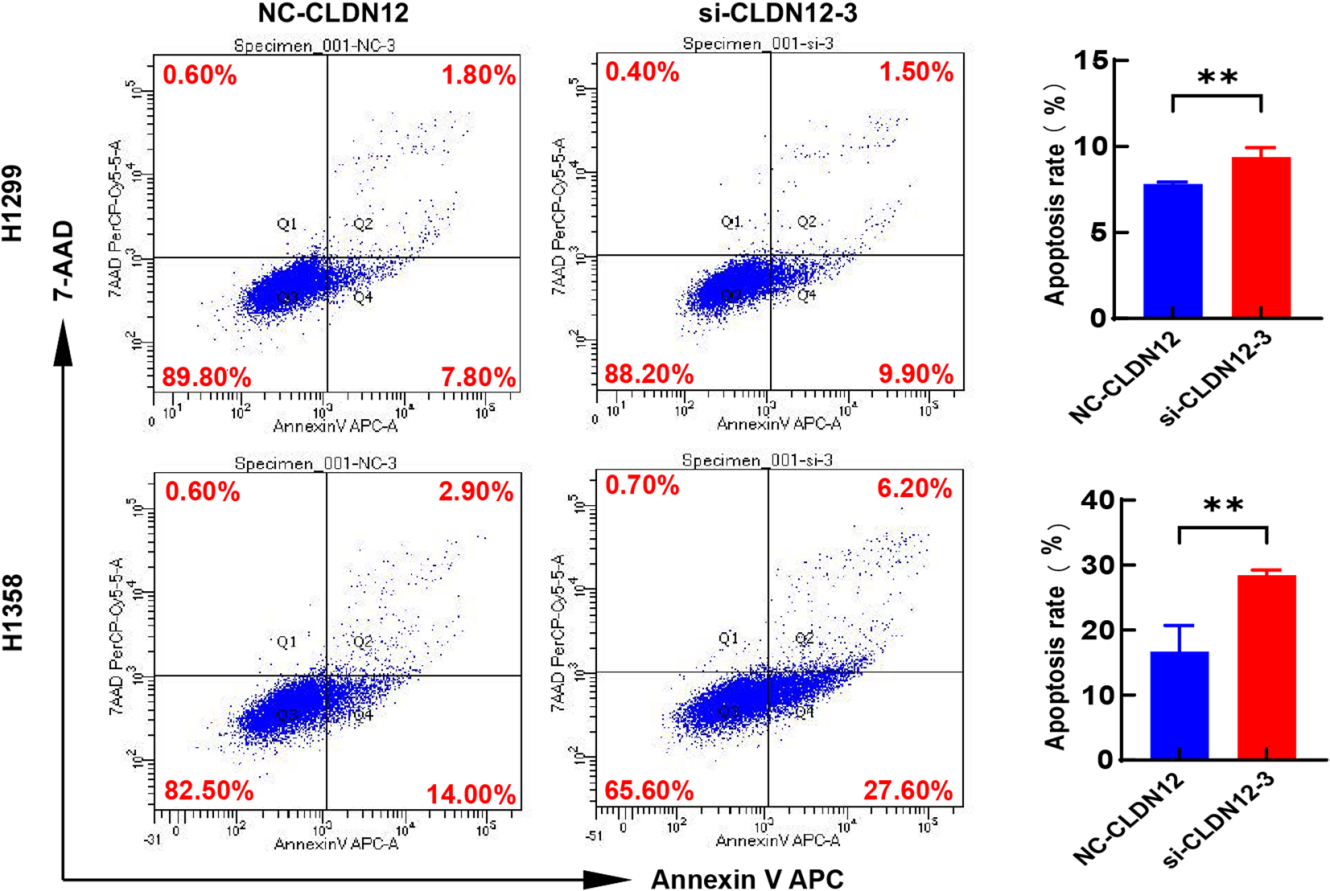
CLDN12 knockdown promoted apoptosis in H1299 and H358 cells. (**P < 0.01) ($\:\stackrel{-}{x}\pm\:s$, *n* = 3). This figure was generated using the GraphPad Prism (version 8.0). Graphic stitching was performed using Adobe Illustrator (version 24.3.0).

A wound healing assay was conducted to assess the impact of CLDN12 knockdown on the cell migration ability. As shown in Fig. 8A and B, when compared to the negative control group, the migration rates of H1299 and H358 cells were remarkably reduced 48 h after the knockdown of CLDN12 (*P* < 0.05). Simultaneously, the Transwell migration assay results also demonstrated that the migration capacity of H1299 and H358 cells was notably diminished following the knockdown of CLDN12 (*P* < 0.05) (Fig. 8C and D). Furthermore, the outcomes of the Transwell invasion assay revealed that the knockdown of CLDN12 led to a significant inhibition of the invasive ability of H1299 and H358 cells (*P* < 0.05) (Fig. 8E and F). Flow cytometry analysis demonstrated that the knockdown of CLDN12 remarkably facilitated the apoptosis of H1299 and H358 cells (*P* < 0.05) (Fig. 9).

## Discussion

Up to now, there exist 27 members of CLDNs in mammals, with their molecular weights spanning from 20 to 34 kDa. Based on the extent of sequence homology, the CLDNs can be classified into two categories: classical (1–10, 14, 15, 17, 19) and non-classical (11–13, 16, 18, 20–27)^18^. Our results showed that CLDNs were expressed in most normal tissues and that the expression of the same CLDNs in different tissues was significantly different. Notably, CLDN12 is expressed in almost all normal tissues, which is consistent with previous studies on the expression of the CLDN gene family^20^. As an important member of TJs, CLDNs play an epithelial barrier protective role through intercellular adhesion. At the same time, CLDNs can regulate the pore size between cells in the epithelial or endothelial barrier and selectively permeate small ions to mediate paracellular movement^30^. CLDNs also endow epithelial cells with polarity and form specific apical domains that are involved in the regulation of various crucial cellular functions^31^.

Not only that, CLDNs play a pivotal role in tumorigenesis and the process of tumor progression. Among these, CLDN1, 4, 6, and 18.2 have already been utilized as targets for cancer treatment^32^. Our findings revealed that nearly all CLDNs exhibited differential expression patterns across various tumor types. CLDN1 was significantly highly expressed in COAD and ESCA, which was confirmed in matched samples from COAD and ESCA patients^33,34^. In TCGA database, CLDN4 was highly expressed in CESC, CHOL, PCPG and endometrial carcinoma (UCEC), and the integration of GTEx database demonstrated that the expression level of CLDN4 was also markedly up-regulated in CESC, CHOL, PCPG and UCEC. This result aligns with previous research on CLDN4 expression and regulation in cancer^35^, which also revealed that reduced CLDN4 expression is a potential marker of EMT.

The majority of the AUC exceeded 0.7, with some even surpassing 0.9. These findings suggest that numerous CLDNs possess certain clinical utility in tumor-related contexts. COX proportional hazards regression univariate analysis of CLDNs in pan-cancer revealed that most CLDNs were independent factors influencing the survival of patients in pan-cancer. The correlation heat map of CLDNs expression in pan-cancer showed that the expression of most CLDNs was positively correlated, among which CLDN4 and 7 had the highest correlation coefficient of 0.89, indicating that CLDNs has a certain combined application value in tumors. The above results collectively demonstrate the wide application value of CLDNs in pan-cancer. For instance, CLDN18.2 has emerged as a distinctive molecular target for the targeted therapy of various cancers^36^, particularly gastric cancer, owing to its specific expression profile in tumor tissues^37^. On this foundation, monoclonal antibodies targeting CLDN18.2, bis-specific antibodies (BsAbs), chimeric antigen receptor T (CAR-T) cells targeting CLDN18.2 and antibody-drug conjugates (ADCs) have been developed and applied in clinical settings^38^.

It has been reported that CLDNs can function as tumor suppressor genes. A study on LUAD^39^ demonstrated that the overexpression of CLDN1 can block the malignant progression of cancer cells. Compared with adjacent normal tissues or gastric epithelial cells, the expression of CLDN4 in gastric cancer tissues and cell lines is reduced. Silencing CLDN4 increases the phosphorylation of PI3K and AKT, and increases the malignant progression of gastric cancer cells^40^. However, accumulating evidence indicates that CLDNs may act as oncogenes in diverse tumor types^41,42^. CLDN1 is strongly expressed in highly aggressive oral squamous cell carcinoma (OSC) cell line OSC-4, and the invasive activity of OSC-4 was significantly inhibited after CLDN1 knockdown^43^. In vivo experiments have confirmed that the CLDN4 exerts a carcinogenic effect in colorectal cancer through the activation of the PI3K/AKT/mTOR signaling pathway^42^. Our analysis also showed that the effects of the same CLDN on different tumors were sometimes opposite. In univariate analysis of COX proportional hazards regression, CLDN1 was a favorable factor for LUAD and STAD, but an unfavorable factor for LAML, ovarian cancer (OV) and thymoma (THYM). In the KM survival curve of CLDN12 and tumors, high expression of CLDN12 was associated with poor prognosis in LUAD, CESC, KICH and LGG, while it was associated with better prognosis in KIRC and LAML. In summary, the same CLDN can have distinct impacts on the biological behaviors of various tumors. Whether it functions as an inhibitor or a promoter hinge upon the tumor type and the diverse interacting molecules within different cells^44^. Overall, the roles of CLDNs in cancer still harbor a vast expanse of uncharted territory awaiting exploration.

According to estimates by the International Agency for Research on Cancer, approximately 20 million new cancer cases emerged globally in 2022, with lung cancer ranking first worldwide^1^. LUAD continues to pose a significant global medical challenge because of its highly malignant features, non-specific symptoms, and low 5-year survival rate. At present, in clinical practice, the treatment of LUAD includes surgery, radiotherapy, chemotherapy, targeted therapy, immunotherapy, or a combination of these treatments^5^. Nevertheless, the emergence of drug resistance and adverse effects associated with chemotherapy, coupled with the restricted indications for targeted therapy and immunotherapy, severely curtail their overall efficacy^45^. Early diagnosis and prompt intervention have been shown to substantially enhance the survival rates of LUAD patients^46^; Consequently, the identification of more specific molecular targets and reliable biomarkers is of paramount importance for the early detection and effective management of LUAD.

Current research focusing on CLDN12 remains relatively scarce, with limited studies investigating this molecule to date. Aside from its low expression in CHOL, KICH, KIRC, and SARC, CLDN12 exhibits high expression in most other cancer types. In-depth exploration of CLDN12 may thus yield valuable insights to advance cancer research in a broader context and ultimately benefit a larger population of cancer patients. For these reasons, we selected CLDN12 as the primary target of our research.

CLDN12 represents a non-classical constituent of the CLDN family. Owing to its lack of a PDZ-binding motif, the functions of CLDN12 diverge somewhat from those of other CLDN members. Firstly, analogous to other classical CLDNs, CLDN12 is a crucial element of TJs. It assumes a significant role in regulating epithelial permeability and providing barrier protection. The difference is that CLDN12, together with CLDN2, can form independent Ca^2+^ osmotic pores in the kidney and colon epithelium, mediating paracellular Ca^2+^ absorption and maintaining Ca^2+^ balance in vivo^22^. Moreover, CLDN12 has different expression and functions in various types of tumors. A study of transplanted tumors (fibrosarcoma, melanoma, and Lewis lung cancer) in Cldn12-deficient mice showed that homotypic interactions between CLDN12 contribute to the migration of myeloid-derived suppressor cells (MDSCS). MDSCs transendothelial migration into tumors and promote tumor growth^47^. CLDN12 has been shown to facilitate the proliferation and migration of osteosarcoma cells via the PI3K/AKT signaling pathway^48^. Additionally, CLDN12 is thought to drive the EMT process of pancreatic cancer cells^49^. However, due to tissue-specific and tumor-specific regulatory mechanisms, decreased expression of CLDN12 predicts poor prognosis of cervical cancer^50^, Interleukin-18 (IL-18) promotes breast cancer cell migration by down-regulating CLDN12 and inducing P38/MAPK pathway^51^, etc.

In this work, public database indicated that CLDN12 was highly expressed in LUAD. This finding is in line with the research outcomes of Xie Bin et al. regarding the relationship between the comprehensive map of linked genes and LUAD patients^52^. The KM survival curve indicated that higher CLDN12 expression levels had a poorer prognosis. The AUC of CLDN12 in LUAD was as high as 0.9311, indicating that the detection method had high authenticity and good prediction effect. The cBioPortal online database was exploited to investigate the potential molecular mechanisms underlying LUAD, and it was found that the frequency of CLDN12 mutation in LUAD patients was as high as 9%. Taking the above results together, it is not difficult to see that CLDN12 has the strongest correlation with LUAD. Next, we focused on the relationship between CLDN12 and LUAD.

In the study of the correlation between CLDN12 and clinical characteristics of LUAD, it can be seen that the expression of CLDN12 in T2-T4 stage and N1-N3 stage is significantly different from that in the corresponding control group, and it is not related to age, gender and distant metastasis. This result is consistent with Li et al. ‘s study on the correlation of clinical features of LUAD^53^. The findings of TCGA, GEO and HPA databases all confirmed that CLDN12 was highly expressed in LUAD. This result lays the foundation for the study of CLDN12 in LUAD.

In the present study, GO functional enrichment and KEGG pathway enrichment analyses were performed on genes associated with CLDN12 expression, utilizing publicly available databases. CLDN12 expression related genes were significantly related to zinc ion transmembrane transport, aminoacyl-tRNA synthetase multi-enzyme complex, COP Ⅱ vesicle capsid cell composition, etc., and were enriched in vascular smooth muscle contraction, p53, Wnt, MAPK and other signaling pathways. Studies have shown that zinc ion metabolism promotes the degradation of extracellular matrix and enhances the invasion ability of tumor cells by regulating the activity of metalloproteinases^54^. Huang et al. confirmed that the CLDN12 tail can bind to Sec24C to reach the cell membrane through the COPII transport system and interact with CD81, thereby promoting the entry of hepatitis C virus into cells^55^. The p53 signaling pathway acts as a key hub in cell cycle regulation, maintaining genomic stability by inducing G1 phase arrest or apoptosis. Mutation or dysfunction of p53 can disrupt cell cycle checkpoints, thereby facilitating unrestricted proliferation of tumor cells^56^. Additionally, activated p53 is capable of suppressing the Wnt signaling pathway, which in turn restrains the growth and invasion of tumor cells. Consequently, we predicted that the functions of CLDN12 in promoting proliferation, enhancing invasion, and inhibiting apoptosis in LUAD cells might be associated with the p53 pathway.

The PPI network, in conjunction with the Cytoscape plugin, was employed to identify hub genes that interact with CLDN12, including HSPA8, HIF-1α, CLTC, DHX15, CXCL8 and WDR36. As a core member of HSP70 family, HSPA8 plays multiple key roles in cell homeostasis maintenance and disease regulation^57^. In the study of CHOL, ribosomal protein L35a (RPL35A) promotes the progression of CHOL by mediating the ubiquitination of HSPA8 protein^58^. HIF-1α is a key transcription factor for maintaining oxygen homeostasis^59^, and its expression level is regulated by hypoxia stimulation^60^. The accelerated proliferation of solid tumors often leads to the formation of a local hypoxic microenvironment. Under hypoxic conditions, the stability of HIF-1α is enhanced, and it translocases to the nucleus to form a heterodimer with HIF-1β, thereby activating downstream target genes and promoting tumor angiogenesis, metabolic reprogramming, and EMT. In various human malignant tumors^61^, including non-small cell lung cancer, the expression level of HIF-1α is elevated and is associated with a poor prognosis^62^. DHX15 can interact with circRNF10 to isolate DHX15 from p65 and inhibit the progression of breast cancer by inhibiting the nuclear factor kappa-light-chain-enhancer of activated B cells (NF-κB) pathway^63^. CLTC is involved in the interaction network of Ras-related protein Rab-7 A in liver cancer, and miRNA-624 promotes the growth of liver cancer cells by regulating this interaction network^64^. In studies of OV and gastric cancer, overexpression of CXCL8 promotes the proliferation, migration, invasion, EMT, and angiogenesis of tumor cells, thereby accelerating peritoneal metastasis^65^.

Interestingly, the outcomes of the GO and KEGG analysis in this study revealed that CLDN12 expression-related genes were closely related to genetic information processing processes such as spliceosome and ubiquitin-mediated proteolysis. Moreover, these genes demonstrated significant enrichment in several types of cancers, including colorectal cancer, breast cancer, and gastric cancer. In conclusion, the identification of the hub genes interacting with CLDN12 has furnished novel insights into deciphering the mode of action of CLDN12 within LUAD. This discovery holds substantial promise and is indeed deserving of thorough and in-depth investigation, as it may potentially unlock new therapeutic targets and strategies for the management of LUAD, thereby enhancing our understanding of the disease’s complex biological processes and ultimately improving patient outcomes.

One of the most significant biological traits of cancer is the speedy and uncontrolled growth and division of cancer cells occurring inside the body. Therefore, the suppression of cancer cell proliferation and the induction of cancer cell apoptosis are of paramount significance in the therapeutic management of diverse malignancies. As previously elaborated, CLDN12 exhibits distinct functions in the processes of proliferation and migration of various cancers, including osteosarcoma, pancreatic cancer, cervical cancer, breast cancer, and others.

Therefore, in this study, we investigated the effects of CLDN12 on the proliferation, migration, invasion, and apoptosis of LUAD cells by silencing CLDN12 expression using siRNA. RT-qPCR analysis demonstrated that the expression level of CLDN12 mRNA in H1299, H358, and H2087 cells was remarkably elevated when compared to that in normal bronchial epithelium BEAS-2B cells (*P* < 0.05). However, no significant disparity was detected in A549 cells. This finding does not fully align with the outcomes of prior bioinformatics analyses. The root cause of this divergence is attributed to the marked heterogeneity in the expression profiles of CLDNs among distinct tissue and cellular contexts, especially when juxtaposing normal and neoplastic tissues. It is plausible that the disparity in CLDNs expression could also be attributed to the generation of distinct isoforms via alternative splicing of transcripts^66^. In this regard, the expression patterns of these isoforms diverge from those of their pre-spliced counterparts. For subsequent experiments, H1299 and H358 cells were chosen. The expression of CLDN12 in these two cell lines was suppressed, and the efficacy of the knockdown was validated through RT-qPCR and WB. In the CCK-8 and colony formation assays, when compared to their respective negative controls, both the cell proliferation capacity and colony formation ability were markedly reduced following CLDN12 knockdown. Meanwhile, CCK-8 and colony formation assays performed using CLDN12-underexpressing and CLDN12-overexpressing stable cell lines further confirmed that CLDN12 underexpression inhibits LUAD cell proliferation, whereas CLDN12 overexpression promotes it. These are consistent with the results of other LUAD proliferation studies^67,68^. Concurrently, flow cytometric analysis revealed that the apoptosis rate was significantly elevated after CLDN12 knockdown. The outcomes of these three experiments collectively indicated that CLDN12 knockdown inhibited the proliferation and promoted apoptosis in the LUAD cell lines H1299 and H358.

Subsequently, the migration and invasion capabilities of CLDN12 were evaluated using the wound healing assay, as well as Transwell migration and invasion assays. Relative to the corresponding negative control cells, the wound healing capacity of CLDN12 knockdown cells was notably diminished 48 h post cell scratching. In the Transwell migration and invasion assays, the migratory and invasive capacities of CLDN12-knockdown cells were significantly suppressed when juxtaposed with the negative control group. These findings solidified the notion that CLDN12 facilitated the migration and invasion of the LUAD cell lines H1299 and H358. Intriguingly, these results mirrored those reported in investigations of CLDN12 in human osteosarcoma and murine transplanted tumors^47,48^, and were congruent with the outcomes of GO and KEGG analysis of CLDN12 expression-associated genes derived from public databases. In summary, the in vitro experiments herein have conclusively demonstrated that CLDN12 promotes the proliferation, migration, and invasion of LUAD cells while concurrently suppressing their apoptosis. These findings are analogous to those from previous in vitro studies that implicated CLDN12 in the regulation of cell migration during the metastatic process^24^.

It is widely recognized that EMT represents a defining characteristic of tumor migration and invasion. During EMT, epithelial cells undergo a phenotypic switch, shedding their epithelial traits and acquiring mesenchymal attributes^69^. Epithelial cells are equipped with diverse intercellular junctional complexes. Among these, CLDNs assume a pivotal role in preserving epithelial polarity and barrier function by virtue of their participation in TJs^15^. CLDNs serve as critical regulators of cell migration, capable of activating key signaling cascades implicated in the EMT process. For instance, CLDN1 has been validated to facilitate the EMT of LIHC by up-regulating Slug and Zeb1^70^. Additionally, IL-18 promotes the migration of breast cancer cells by downregulating CLDN12 and triggering the P38/ MAPK pathway^51^, and so on. In the process of EMT, adherent junctions are composed of E-, N-, R-, and P-cadherins^15^. These cadherins are classified as type I transmembrane adhesion proteins, and their functionality is contingent upon the presence of extracellular Ca^2+^. In this study, GO functional enrichment showed that genes related to CLDN12 expression were associated with cadherin binding function. Therefore, we hypothesized that CLDN12 may promote EMT in LUAD cells by binding to cadherin and is closely related to the effect of Ca^2+^. Similar to the results of our analysis, CLDN12 was confirmed to be expressed in the proximal tubule and confer paracellular calcium permeability^71^. Recent investigations have revealed that colostrum basic protein enhances calcium absorption in mice by up-regulating the expression of calcium transporter paracellular proteins CLND2 and CLND12. This upregulation subsequently increases bone mineral density and stimulates bone growth^72^. Collectively, these studies underscore the pivotal role of CLDN12 in modulating Ca^2+^ uptake and Ca^2+^ signaling cascades. Moreover, the calcium signaling pathway promotes cancer cell migration, invasion, and chemoresistance by regulating calmodulin-dependent kinases and calcium ion channels^73^. Therefore, as a transmembrane protein, CLDN12 may modulate the metastatic ability of tumor cells through regulating calcium influx, or by interacting with cadherins and influencing calcium-dependent signal transduction pathways.

The research on LUAD has long been a focal point for numerous scholars. Certain studies center on the development of learning models for the early detection of LUAD, employing omics-based approaches^74,75^. Other investigations utilize the genes with differential expression in LUAD from databases such as TCGA and the GEO to construct morphological maps, aiming to elucidate their associations with LUAD^52^. Our study, however, not only involved the comparison and screening of differentially expressed genes between LUAD tissues and normal lung tissues by leveraging multiple databases. Through this, we analyzed the relationships among the expression of CLDN12 in LUAD, the prognosis of patients, and the clinical stages of the disease. Additionally, we experimentally confirmed in vitro that CLDN12 promotes the proliferation, migration, and invasion of LUAD cells, while simultaneously inhibiting their apoptosis. Moreover, we analyzed the functions and potential pathways of crucial genes related to CLDN12 expression, including HSPA8 and HIF-1α, in the process of tumor progression. This analysis offers novel perspectives for the in-depth exploration of LUAD and the identification of reliable biomarkers. Nonetheless, this work is not without its limitations. We have only conducted a preliminary investigation into CLDN12. In subsequent studies, we will further elaborate on the mechanism of action of CLDN12 and its potential value in pan-cancer research through both in vivo and in vitro experiments.

## Conclusion

The findings of this study indicate that CLDNs exhibit differential expression across various cancers and possess both prognostic and diagnostic significance in oncology. CLDN12 is up-regulated in LUAD, and its expression is correlated with the tumor stage and lymph nodes metastasis in LUAD patients. A high level of CLDN12 expression is linked to a poor prognosis for LUAD patients. Functionally, CLDN12 promotes the proliferation, migration, and invasion of LUAD cells while suppressing apoptosis. Consequently, CLDN12 has the potential to serve as a biomarker for the diagnosis and prognosis assessment of LUAD. This discovery provides a theoretical basis for the early detection and treatment of LUAD. However, the underlying mechanisms by which CLDN12 exerts these effects require further in-depth investigation and exploration.

## Materials and methods

### Data information

For pan-cancer analysis, the gene expression matrix of 7,775 normal tissues was extracted from the GTEx database (https://gtexportal.org), whereas the mRNA expression matrix of 10,530 cancer and paracancerous tissue samples was derived from the TCGA database (https://cancergenome.nih.gov). All these matrices, as well as the corresponding clinical information, were downloaded after standardization by the University of California, Santa Cruz (UCSC) Xena database (https://xenabrowser.net). LUAD patient datasets GSE87410, GSE102511, GSE116959 and GSE140343 were obtained from the GEO database (https://www.ncbi.nlm.nih.gov/geo). The dataset GSE87410^76^ consisted of 5 pairs of samples (tumor tissue from LUAD and normal lung tissue). The dataset GSE102511^77^ consisted of 31 samples (16 tumor tissues from LUAD and 15 normal lung tissues). The dataset GSE116959^78^ consisted of 68 samples (57 tumor tissues from LUAD and 11 normal lung tissues). The dataset GSE140343^79^ consisted of 100 samples (51 tumor tissues from LUAD and 49 non-cancerous adjacent tissues). The IHC images were retrieved from the HPA database (https://www.proteinatlas.org). We selected one normal tissue sample and four LUAD tissue samples-from patients including both males and females-as representative images for presentation. Normal tissue sample: ID 2101, male. LUAD tissue samples: ID 1847, male; ID 2567, male; ID 3048, female; ID 3052, female. We used the cBioportal database (http://www.cbioportal.org) to analyze the relationship between the occurrence of LUAD and CLDNs mutations.

### Bioinformatics analysis

R (version 4.3.0) and associated R packages were used. Differential gene analysis was performed using the DESeq2 package^80^ (version 1.46.0). “*P* < 0.05 and log_2_FC (Fold Change, FC) ≥ 2” was defined as the screening criteria for differential mRNA expression. Heatmaps were drawn using the ComplexHeatmap package^81^ (version 2.22.0). ROC was drawn and analyzed by pROC package (version 1.18.5). Pearson correlation analysis was used to analyze the correlation among CLDNs, and then heatmaps were drawn with the corrplot package (version 0.95). KM survival analysis, univariate COX regression analysis and graph drawing were performed using the survival package (version 3.5-7) and the survminer package (version 0.4.9). All bar graphs were drawn using the ggplot2 package (version 3.5.1).

A total of 854 genes demonstrated significant co-expression with CLDN12, as determined by correlation analysis (significance cutoff: *P* < 0.01; correlation strength: |R| ≥ 0.3). GO and KEGG^82^ analyses were performed using clusterProfiler^83^ (version 4.14.4) and enrichplot packages (version 1.26.2). The STRING database (https://cn.string-db.org) was used to establish the PPI network targeting CLDN12. After setting the parameter as “medium confidence = 0.4”, the PPI network was put into Cytoscape (version 3.7.0). Using the Cytoscape plugin MCODE (version 1.6.1), the node degree threshold was set ≥ 2.0, the node with only one edge connected was removed, the node score threshold was ≤ 0.2, and the K value was ≥ 2.0 to obtain the CLDN12 PPI subnetwork, and the subnetwork with the highest score was selected for further analysis. The PPI network was analyzed using the CytoHubba plugin (version 0.1). Nine topology algorithms integrated within the CytoHubba plugin were utilized to generate a core gene set. Each algorithm contributed its top 10 genes to this set. The gene sets identified as core genes by 3 or more algorithms were intersected with the maximum subnetwork obtained by the MCODE plugin to obtain 6 hub genes.

### Cell culture

Human normal bronchial epithelium cells BEAS-2B were purchased from Shanghai Lianmai Life Technology Co., Ltd. LUAD cell lines A549, H358, H1299, H1650, and H2087 were obtained from Wuhan Punuosai Life Technology Co., Ltd. The BEAS-2B cells were cultivated in Dulbecco’s Modified Eagle’s Medium (DMEM) with high glucose content, which was supplemented with 20% fetal bovine serum (FBS) and 1% penicillin/streptomycin. Conversely, A549 cells were grown in Ham’s F-12 K medium containing 10% FBS and 1% penicillin/streptomycin. The H358, H1299, H1650, and H2087 cell lines were cultured in Roswell Park Memorial Institute (RPMI) 1640 medium, which was fortified with 10% FBS and 1% penicillin/streptomycin. All cell cultures were maintained at 37 °C under an atmosphere of 5% CO₂. The cells used in the experiments were all in the exponential growth phase after three stable passages.

### RNA extraction and RT-qPCR

Total cellular RNA was extracted using TransZol UP following the manufacturer’s instructions provided in the RNA extraction kit (Quanshijin ER501). The NanoDrop ND-2000 was employed to assess the concentration and purity of RNA samples. A one-step reverse transcription kit (Quanshijin AE311) was utilized to convert total RNA into cDNA. According to the reverse transcription quantitative PCR kit (Quanshijin AQ601), the reaction parameters were set as follows: 94℃ for 30 s, 94℃ for 5 s, 60℃ for 30 s, 40–45 cycles. β-actin was used as an internal reference for calculations using 2^−∆∆Ct^ and the sequences of gene-specific primers are shown in Table 1.

**Table 1:** Primer sequences for RT-qPCR.

Gene	Forward (5’-3’)	Reverse (3’ -5’)
β-actin	CCTGGCACCCAGCACAAT	GGGCCGGACTCGTCATAC
CLDN12 si-CLDN12-1 si-CLDN12-2	CAGCCACAGTCCTTTCCTTC GCCUGAUGUACGACACUACUUTT CCCAUCUAUCUGGGUCAUCUUTT	AGTCACTGCTCCCGTCATAC AAGUAGUGUCGUACAUCAGGCTT AAGAUGACCCAGAUAGAUGGGTT
si-CLDN12-3	CAGUAGUUUCACACACCACUUTT	AAGUGGUGUGUGAAACUACUGTT
NC-CLDN12	UUCUCCGAACGUGUCACGUTT	ACGUGACACGUUCGGAGAATT

### Protein extraction and WB

β-actin was utilized as the internal reference control for WB. Proteins were extracted from cells using cell lysis buffer, and protein concentration was determined with the BCA Protein Assay Kit (Solarbio). Sodium dodecyl sulfate-polyacrylamide gel electrophoresis (SDS-PAGE) was performed at a constant voltage of 120 V for 20 min, followed by transfer at a constant current of 400 mA for 20 min. After transfer, the membrane was placed in a 5% skim milk solution and incubated at room temperature for 60 min for blocking. The membrane was then thoroughly rinsed with Tris-buffered saline with Tween-20 (TBST) and incubated with the primary antibody (β-actin: Affinity, lot number: AF7018; CLDN12: Affinity, lot number: DF9390) overnight at 4 °C. Following another rinse with TBST, the membrane was incubated with a goat anti-rabbit IgG secondary antibody (Bioss, lot number: BE02187352) at room temperature for 60 min. After a final rinse with TBST, ECL chemiluminescent solution was prepared; the membrane was evenly immersed in the solution, and exposure was performed to acquire images. The gray values of the protein bands were analyzed using ImageJ software.

### Cell transfection

si-CLDN12 was employed to specifically knockdown CLDN12 expression, while NC-CLDN12 served as the corresponding negative control. 4 × 10^5^ cells in logarithmic growth phase were seeded in culture dishes. The next day, the transfection reagent (Sangon Biotech, 607402) was added to the culture dishes. 24 h after transfection, the cells were collected to extract total RNA, and RT-qPCR was used to detect the knockdown effect. NC-CLDN12 and si-CLDN12 sequences are shown in Table 1.

The lentiviruses used in this study were as follows: CLDN12 underexpression lentivirus: sh-CLDN12 (Catalog NO.: LVP0699-2-4; Titer: 2.5 × 10⁸ TU/mL) and its negative control (NC-sh, Vector: pLO402; Titer: 2.5 × 10⁸ TU/mL); CLDN12 overexpression lentivirus: OE-CLDN12 (Catalog NO.: LVP0698-1-2; Titer: 2.5 × 10⁸ TU/mL) and its negative control (NC-OE, Vector: pLO301; Titer: 2.5 × 10⁸ TU/mL). All lentiviruses were synthesized by Beijing Tsingke Biotechnology Co., Ltd.

When cells reached the logarithmic growth phase, they were digested with trypsin, resuspended, and adjusted to a density of 1 × 10⁵ cells/mL. The cell suspension was inoculated into 6-well plate at 2 mL per well, and the plates were incubated overnight in a 37 °C, 5% CO₂ incubator to allow complete cell adherence. Based on the optimal multiplicity of infection of the lentivirus, the required volume of lentivirus was calculated. A transfection system was prepared using medium without penicillin/streptomycin (90% basal medium + 10% FBS), which was then added to the 6-well plate. After gentle mixing, the plates were returned to the incubator for further culture. At 48 h post-transfection, cell status was observed under a fluorescence microscope to assess transfection efficiency. Puromycin was then added for selection, and selection was continued for 10 days until all uninfected cells had completely detached. Following selection, the puromycin concentration was adjusted to the maintenance level for subsequent culture.

### Cell proliferation

Cell proliferation assay was performed using CCK-8. The cells were cultured in RPMI 1640 medium supplemented with 10% FBS and 1% penicillin/streptomycin. They were seeded into 96-well plates at a density of 8 × 10³ cells per well. The cells were cultured in a 5% CO_2_ cell incubator at 37 °C until they were completely adherent. After 0, 24, and 48 h of culture, 10 µL CCK-8 reagent was added to each well and incubated for 1 h to measure the absorbance value at 450 nm.

### Clone formation

After seeding the cells in 6-well plates at a density of 2 × 10^3^ cells per well, they were cultured in a 5% CO₂ cell incubator at 37 °C for 24 h. Then, the cells were transfected with NC-CLDN12 and si-CLDN12-3, respectively. After 24 h of culture, the complete medium was replaced. Subsequently, the medium was changed every two days, and the culture was terminated when macroscopic single-cell clones were formed. Cells were washed twice with precooled Phosphate Buffered Saline (PBS), fixed with 4% paraformaldehyde for 20 min, stained with 0.1% crystal violet for 15 min, rinsed gently with running water, and dried in air. Images were collected for colony counting and further analysis.

### Wound healing

Before the experiment, five parallel lines through the well plate were drawn on the back of the 6-well plate using a marker. Cells were seeded in 6-well plates at 8 × 10^5^ cells per well and cultured in a 5% CO_2_ cell incubator at 37 °C for 24 h. When the cell confluence reached more than 90%, a scratch was made on the cell layer using the tip of a 200 µL sterile pipette, ensuring that the scratch was perpendicular to the transverse lines at the bottom of the well plate. Then, the wound was washed twice with PBS, and NC-CLDN12 and si-CLDN12-3 were added respectively. Pictures were taken at 0, 24, and 48 h to record the wound healing, and Image J software was used to analyze the scratch images.

### Transwell cell migration

100 µL RPMI 1640 medium was added to the upper chamber of the Transwell chamber, and the cells in the logarithmic growth phase were cultured in serum-free medium for 24 h to prepare a suspension containing 2.5 × 10^4^ cells. The 100 µL cell suspension (containing 20 µL NC-CLDN12 or si-CLDN12-3) was uniformly seeded in the upper chamber of the chamber, and 600 µL medium containing 10% FBS was added to the lower chamber and cultured in a 5% CO_2_ cell incubator at 37 °C. After 24 h, the medium in the chamber was discarded, fixed with 4% paraformaldehyde for 20 min and stained with 0.1% crystal violet for 15 min, and the cells in the upper chamber were wiped off with cotton swabs and allowed to air dry. Pictures were taken under the microscope and saved, and the experimental results were processed with Image J and analyzed.

### Transwell cell invasion

One day prior to cell inoculation, the Matrigel (Corning,356234) was retrieved from the − 20 °C refrigerator and allowed to thaw in the 4°C refrigerator overnight. The next day, after the Matrigel was evenly mixed with serum-free RPMI 1640 medium at a ratio of 1:8, 100 µL of diluted Matrigel was added to the upper chamber of a Transwell chamber and placed in a cell incubator at 5% CO_2_ at 37 °C for 3 h. After 3 h, the 24-well plate was removed from the incubator, the excess matrix gel in the upper chamber of the Transwell chamber was aspirated and discarded, and 100 µL RPMI 1640 medium was added to each chamber and placed in the cell incubator at 5% CO_2_ for 30 min at 37 °C for hydration. Cells in the logarithmic growth phase were cultured in serum-free medium for 24 h to prepare a suspension containing 2.5 × 10^4^ cells. 100 µL cell suspension (containing 20 µL NC-CLDN12 or si-CLDN12-3) was uniformly seeded in the upper chamber, and 600 µL medium containing 10% FBS was added to the lower chamber. The cells were cultured in a 5% CO_2_ cell incubator at 37 °C. After 24 h, the medium in the chamber was discarded, fixed with 4% paraformaldehyde for 20 min and stained with 0.1% crystal violet for 15 min, and the cells in the upper chamber were wiped off with cotton swabs and allowed to air dry. The bottom surface of the upper chamber was removed, sealed with neutral gum and allowed to dry, and then photographed under a microscope for preservation. The experimental results were analyzed after processing with Image J (version 1.8.0).

### Cell apoptosis

Annexin V-APC/7-AAD staining experiment (Elabscience, lot number: E-CK-A218). The cells in logarithmic growth phase were washed with PBS and digested with trypsin without ethylenediaminetetraacetic acid to prepare a suspension containing 5 × 10^5^ cells. The supernatant was discarded by centrifugation, rinsed once with PBS, centrifuged and resuspended in 500 µL binding buffer. 5 µL of Annexin V-APC and 5 µL of 7-AAD were added for staining in the dark for 20 min. Flow cytometry was performed using the BD LSRFortessa cell analyzer and analyzed using FlowJo (version 10.10.0) software.

### Data statistics

All data were analyzed using GraphPad Prism (version 8.0), and the results were expressed as mean ± standard deviation ($\:\stackrel{-}{x}\pm\:s$). Each group of experiments was repeated three times. A paired-sample or independent-sample t-test was used for comparison between two groups. For comparisons among multiple groups, a homogeneity-of-variance test was first performed. If the variances were homogeneous, one-way ANOVA was used; if the variances were heterogeneous, the rank-sum test was used. *P* < 0.05 was considered statistically significant. All figures were edited in Adobe Illustrator (version 24.3.0).

## Supplementary Information

Below is the link to the electronic supplementary material.

Supplementary Material 1

## Data Availability

The datasets employed for analysis in this study can be accessed from the following databases: GTEx (https://gtexportal.org), TCGA (https://cancergenome.nih.gov), UCSC Xena (https://xenabrowser.net), HPA (https://www.proteinatlas.org), cBioportal (http://www.cbioportal.org), and STRING (https://cn.string-db.org). LUAD patient datasets GSE87410, GSE102511, GSE116959 and GSE140343 were obtained from the GEO database (https://www.ncbi.nlm.nih.gov/geo).

## Abbreviations

ACC: Adrenocortical carcinoma
ADCs: Antibody-drug conjugates
AUC: Areas under the curve
BLCA: Bladder urothelial carcinoma
BRAF: B-Raf proto-oncogene
BRCA: Breast invasive carcinoma
BsAbs: Bis-specific antibodies
CAR-T: Chimeric antigen receptor T cells
CAV-1: Caveolin-1
CCK-8: Cell counting kit-8
CESC: Cervical squamous cell carcinoma and endocervical adenocarcinoma
CHOL: Cholangiocarcinoma
CLDN12: Claudin12
CLDNs: Claudins
CLTC: Clathrin heavy chain
CMA: Chaperone-mediated autophagy
COAD: Colon adenocarcinoma
COP II: Coat protein complex II
CXCL8: C-X-C motif chemokine ligand 8
DHX15: DEAH-box helicase 15
DLBC: Lymphoid neoplasm diffuse large B-cell lymphoma
DMEM: Dulbecco’s Modified Eagle’s Medium
EMT: Epithelial mesenchymal transition
ESCA: Esophageal carcinoma
FBS: Fetal bovine serum
GBM: Glioblastoma multiforme
GEO: Gene expression omnibus
GO: Gene ontology
GTEx: Genotype-tissue expression
HIF-1α: Hypoxia-inducible factor 1α subunit
HNSC: Head and neck squamous cell carcinoma
HPA: The human protein atlas
HSPA8: Heat shock protein family A (Hsp70) member 8
IL-18: Interleukin-18
IHC: Immunohistochemistry
KEGG: Kyoto encyclopedia of genes and genomes
KM: Kaplan-Meier
KICH: Kidney chromophobe
KIRC: Kidney renal clear cell carcinoma
KIRP: Kidney renal papillary cell carcinoma
LAML: Acute myeloid leukemia
LGG: Brain lower grade glioma
LIHC: Liver hepatocellular carcinoma
LUAD: Lung adenocarcinoma
LUSC: Lung squamous cell carcinoma
MDSCs: Myeloid-derived suppressor cells
MESO: Mesothelioma
NF-κB: Nuclear factor kappa-light-chain-enhancer of activated B cells
OSC: Oral squamous cell carcinoma
OV: Ovarian serous cystadenocarcinoma
PAAD: Pancreatic adenocarcinoma
PBS: Phosphate buffered saline
PCPG: Pheochromocytoma and paraganglioma
PPI: Protein-protein interaction
PRAD: Prostate adenocarcinoma
READ: Rectum adenocarcinoma
ROC: Receiver operating characteristic
RPL35A: Ribosomal protein L35a
RPMI: Roswell Park Memorial Institute
RT-qPCR: Reverse transcription quantitative polymerase chain reaction
SARC: Sarcoma
SDS-PAGE: Sodium dodecyl sulfate-polyacrylamide gel electrophoresis
SKCM: Skin cutaneous melanoma
STAD: Stomach adenocarcinoma
TBST: Tris-buffered saline with Tween-20
TCGA: The cancer genome atlas
TGCT: Testicular germ cell tumors
THCA: Thyroid carcinoma
THYM: Thymoma
TJs: Tight junctions
UCEC: Uterine corpus endometrial carcinoma
UCS: Uterine carcinosarcoma
UCSC: University of California, Santa Cruz
UVM: Uveal melanoma
WB: Western blotting
WDR36: WD repeat domain 36
