# Supplementary material for: The claudin family characteristics in pan-cancer and the role of claudin12 in the malignant progression of lung adenocarcinoma
Source: Sci Rep. 2025 Dec 29;15:44686. doi: 10.1038/s41598-025-28317-7 (PMC12749535; doi:10.1038/s41598-025-28317-7)
Supplement: Supplementary file 1 — Supplementary Material 1 [file 41598_2025_28317_MOESM1_ESM.pdf]

The claudin family characteristics in pan-cancer and the role of claudin12 in the malignant progression of lung adenocarcinoma

Supplementary materials

Pei Gao[1], Yan Wu[1], Yuxin Bian[2], Yixuan Liu[1], Weixin Jing[1, 3, *]

1.Department of Biology, School of Basic Medical Sciences, Xinjiang Medical University, Urumqi 830017, China;

2.Clinical Laboratory Center, the Fourth Affiliated Hospital of Xinjiang Medical University, Urumqi 830001, China;

3.Xinjiang Key Laboratory of Molecular Biology for Endemic Diseases, Xinjiang Medical University, Urumqi 830017, China.

*: Corresponding author,

Weixin Jing,

E-mail: jing@xjmu.edu.cn

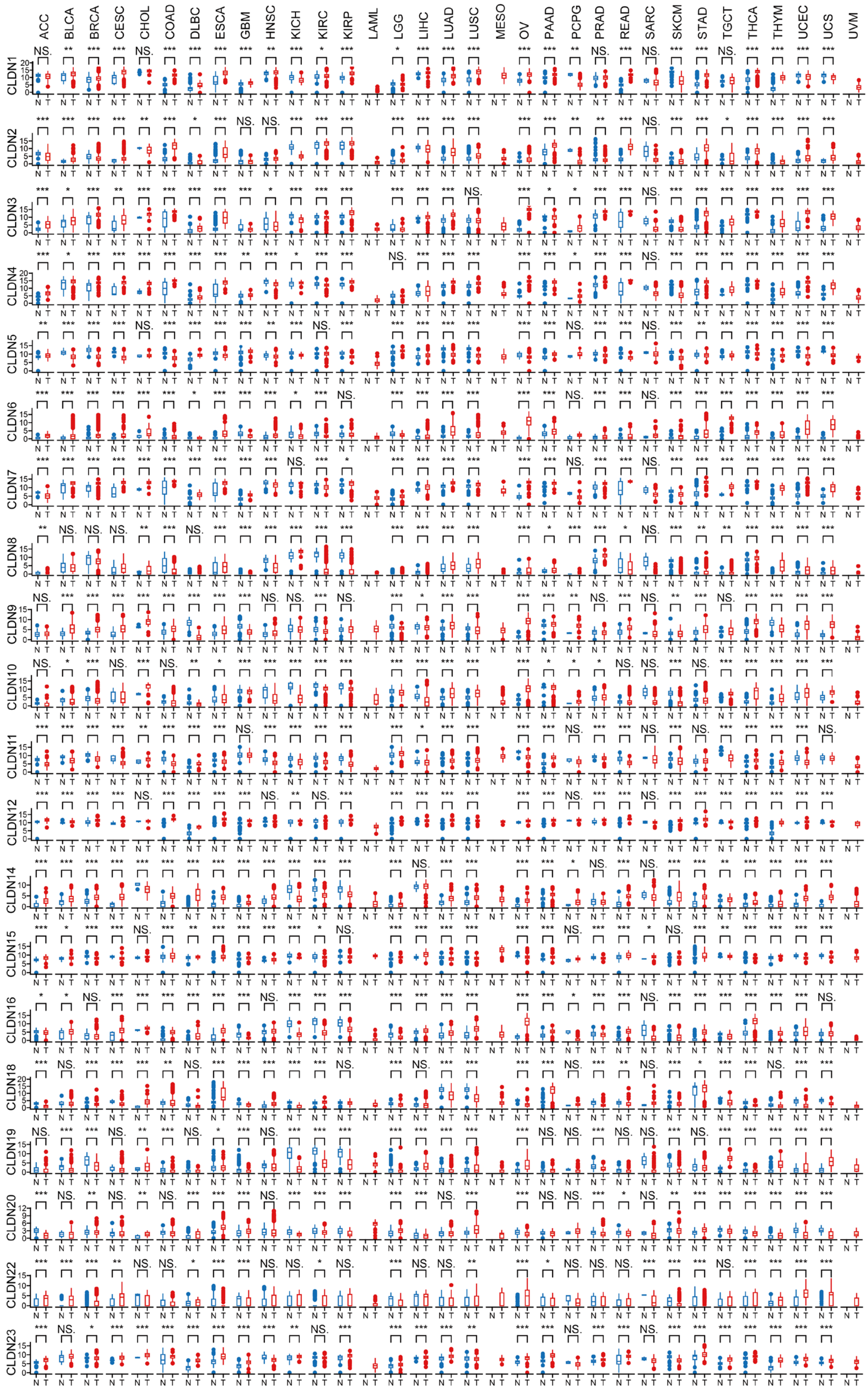

Figure S1 Expression of CLDNs in pan-cancer in GTEx and TCGA databases; N: normal tissue, T: tumor tissue. (**P* < 0.05, ***P* < 0.01, ****P* < 0.001)

This figure was generated using the ggplot2 (version 3.5.1) in R (version 4.3.0). Graphic stitching was performed using Adobe Illustrator (version 24.3.0).

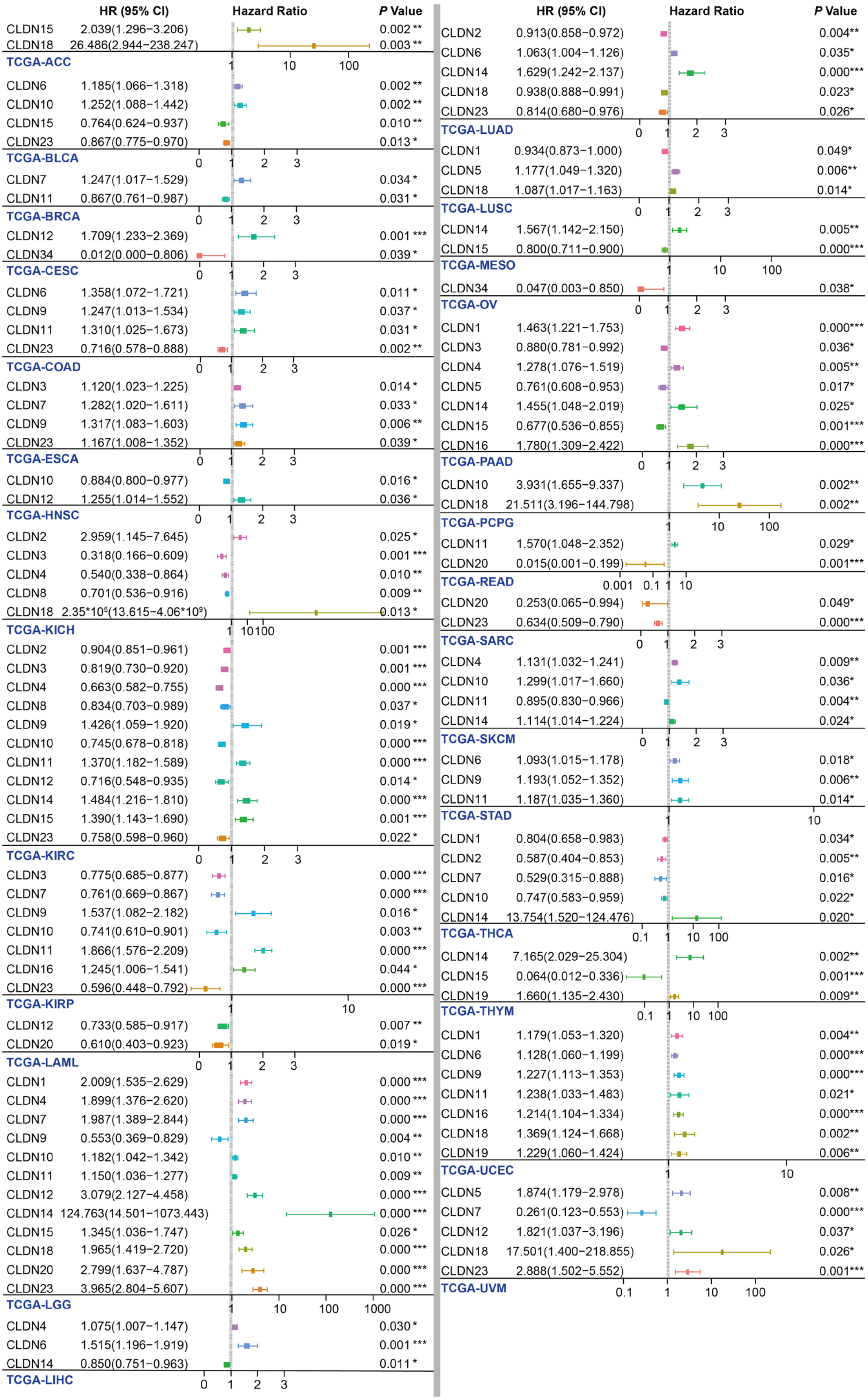

Figure S2 COX proportional hazards regression analysis of CLDNs in pan-cancer. (**P* < 0.05, ***P* < 0.01, ****P* < 0.001)

This figure was generated using the survminer package (version 0.4.9) in R (version 4.3.0). Graphic stitching was performed using Adobe Illustrator (version 24.3.0).

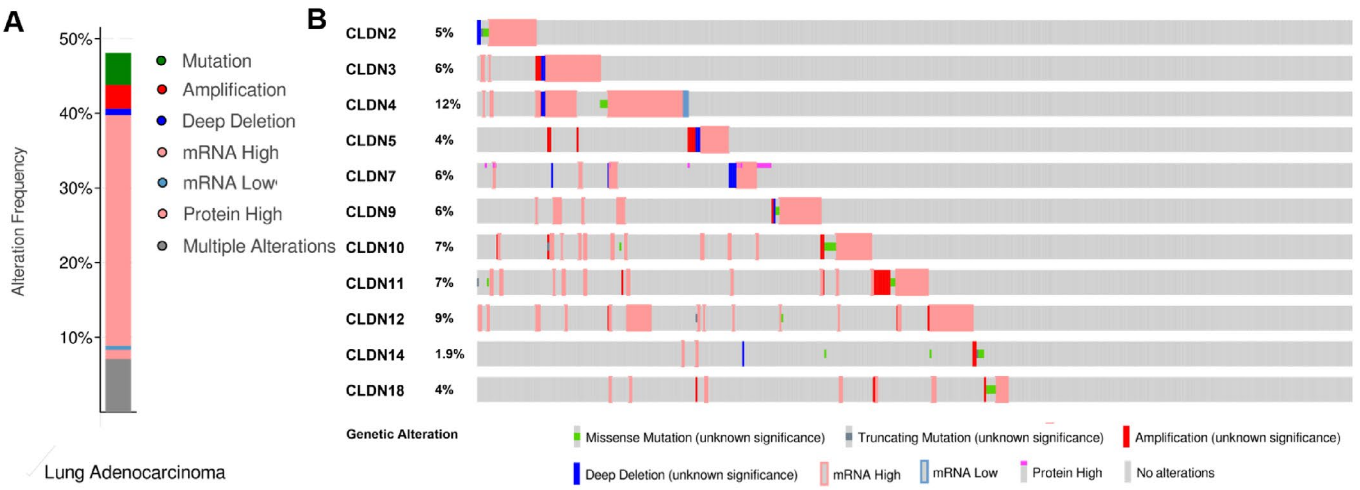

Figure S3 Relationship between the occurrence of LUAD and CLDNs variants in the cBioPortal online database.

This figure was generated using the cBioportal database (http://www.cbioportal.org).

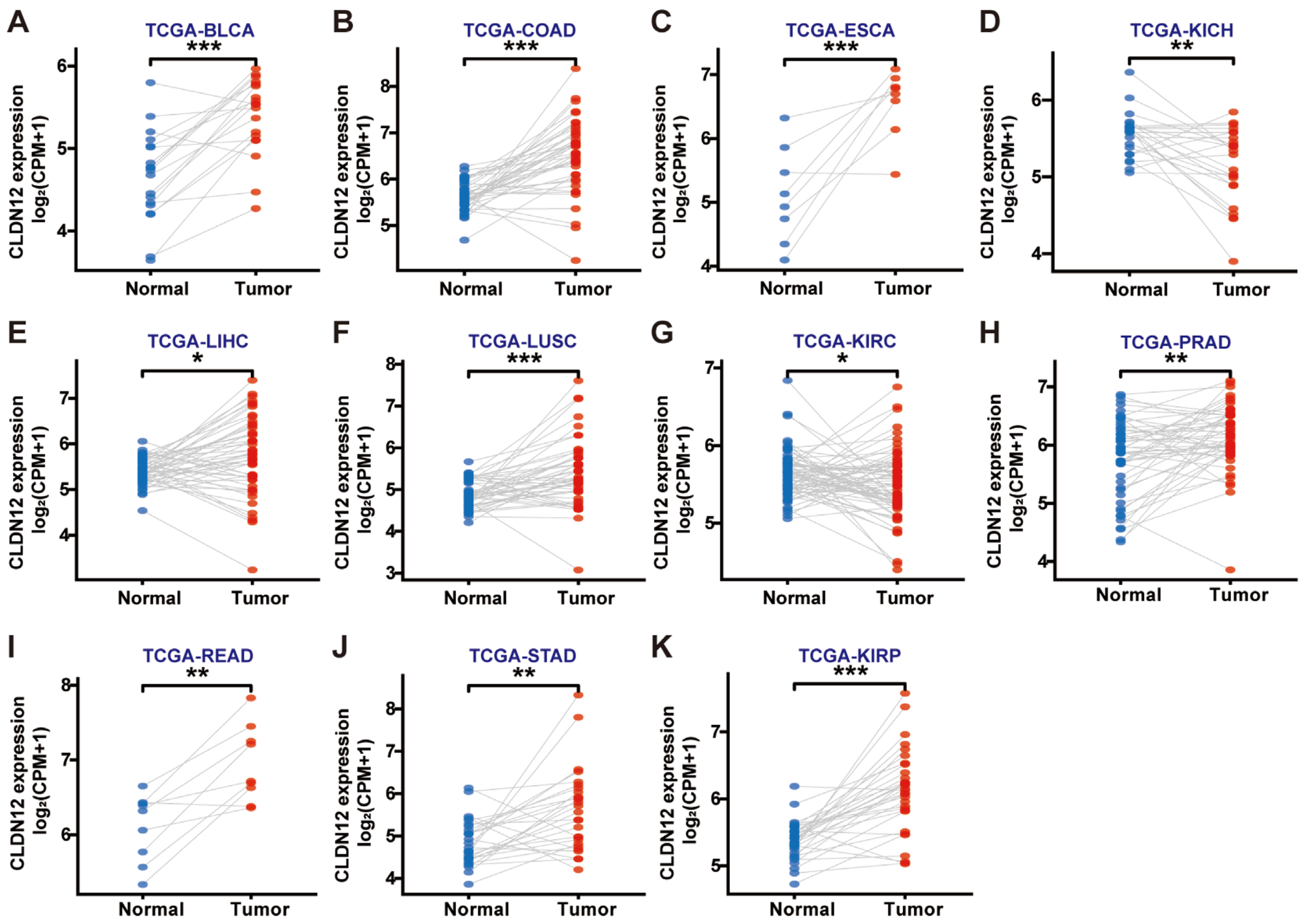

Figure S4 Expression of CLDN12 in paired tumor samples from TCGA database. (*$P < 0.05$, **$P < 0.01$, ***$P < 0.001$)

This figure was generated using the ggplot2 (version 3.5.1) in R (version 4.3.0). Graphic stitching was performed using Adobe Illustrator (version 24.3.0).

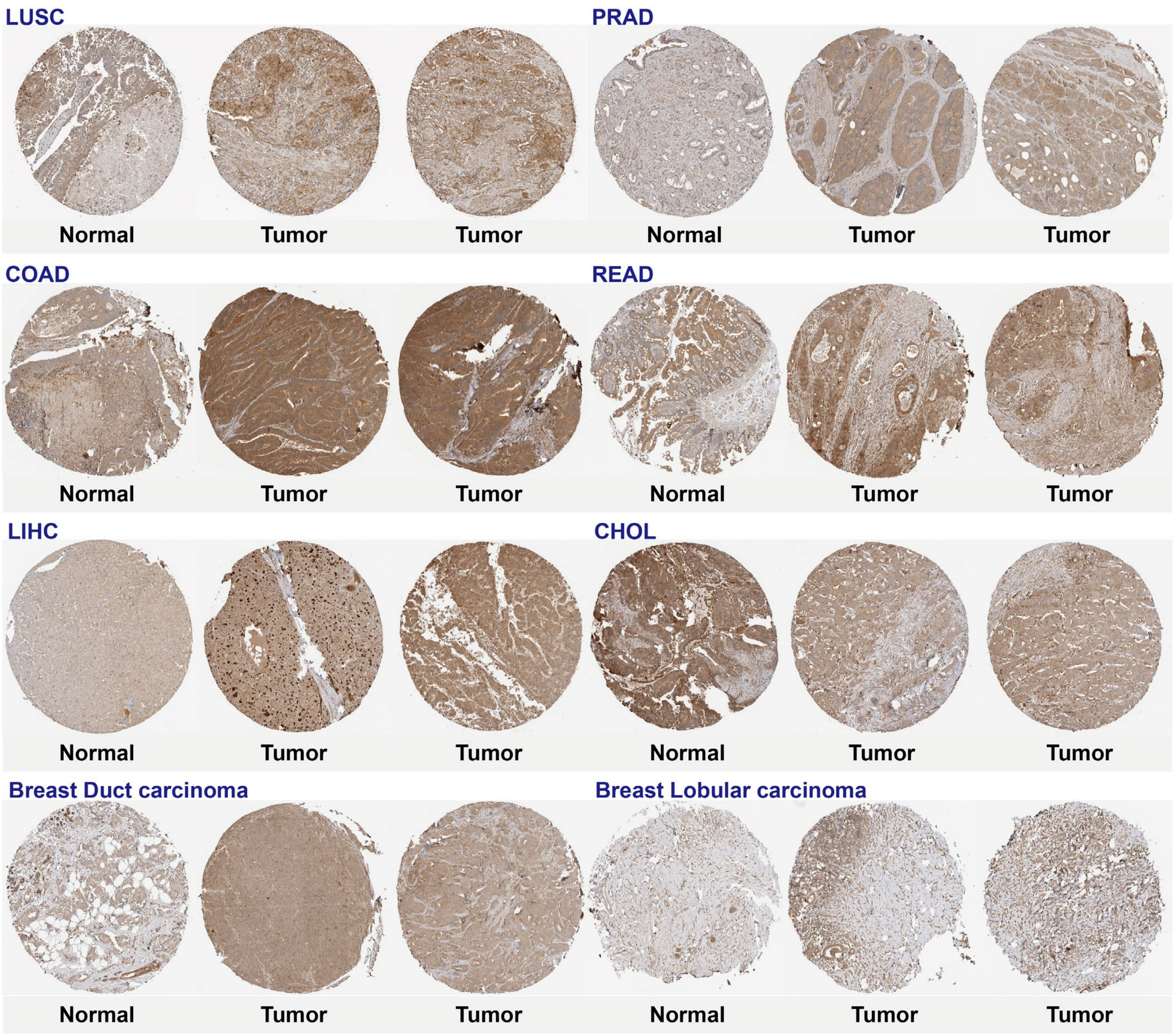

Figure S5 CLDN12 expression in tumor samples and normal tissues from the HPA database (https://www.proteinatlas.org).

This figure was edited in Adobe Illustrator (version 24.3.0).

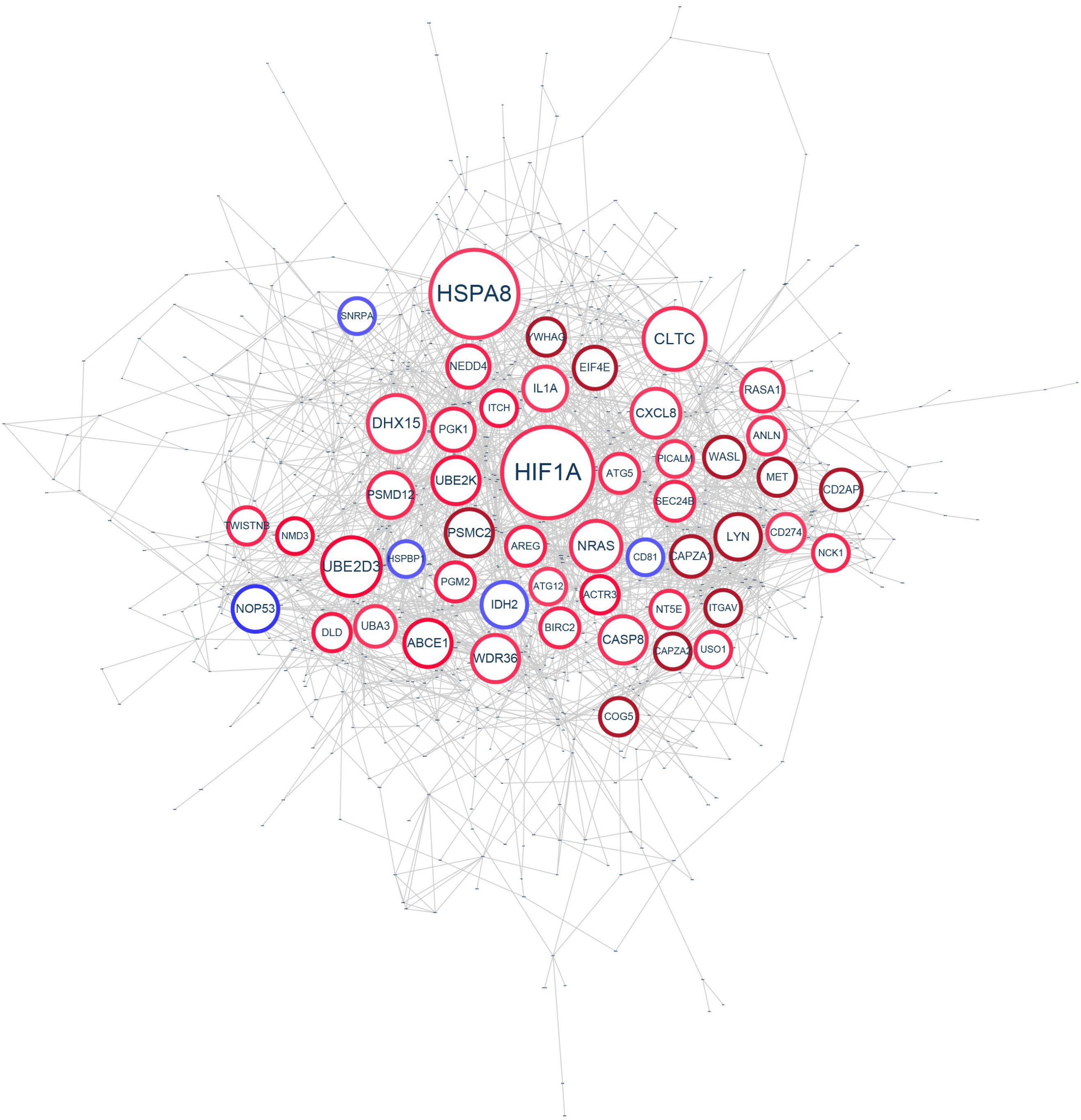

Figure S6 Graph of the PPI network targeting CLDN12.

This figure was generated using the MCODE plugin (version 1.6.1) in Cytoscape (version 3.7.0).

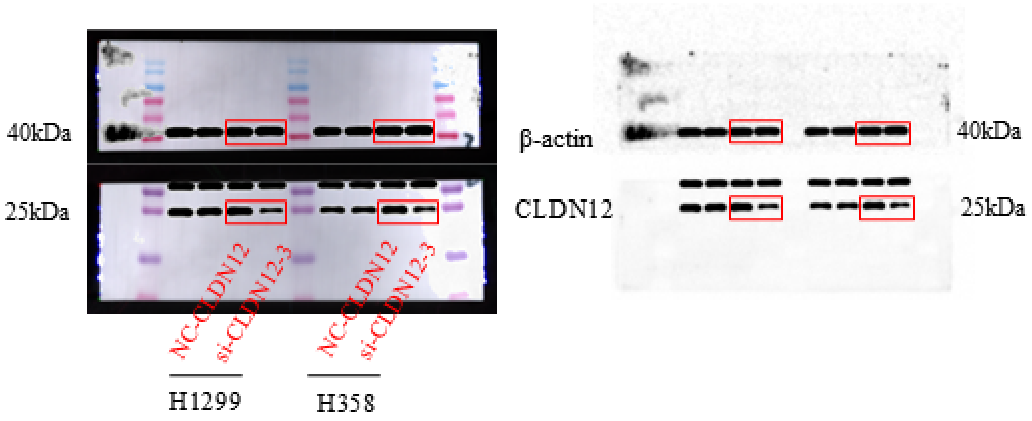

Figure S7 Original Western Blots corresponding to Figure 6D and E.

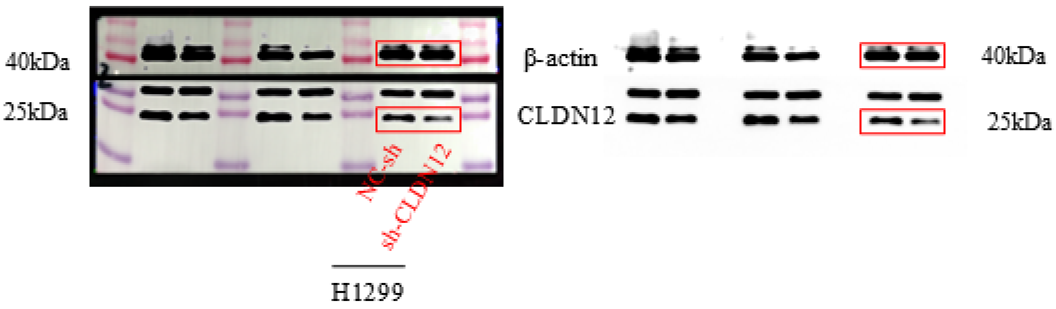

Figure S8 Original Western Blots corresponding to Figure 7B.

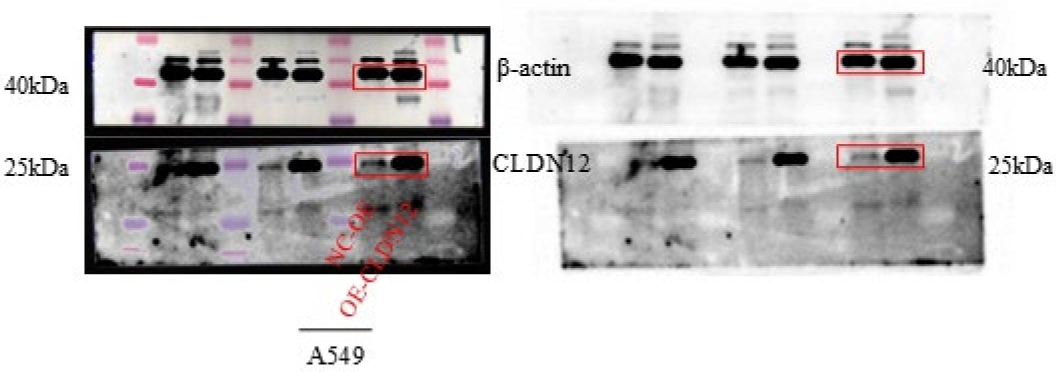

Figure S9 Original Western Blots corresponding to Figure 7D.
